# Intelligent phototriggered nanoparticles induce a domino effect for multimodal tumor therapy

**DOI:** 10.7150/thno.55708

**Published:** 2021-04-19

**Authors:** Xiao Xu, Chao Han, Can Zhang, Dan Yan, Chunling Ren, Lingyi Kong

**Affiliations:** 1 State Key Laboratory of Natural Medicines, Jiangsu Key Laboratory of Bioactive Natural Product Research, School of Traditional Chinese Pharmacy, China Pharmaceutical University, 24 Tong Jia Xiang, Nanjing 210009, PR China.; 2 State Key Laboratory of Natural Medicines, Center of Drug Discovery and Department of Pharmaceutics, China Pharmaceutical University, 24 Tong Jia Xiang, Nanjing 210009, PR China.

**Keywords:** multimodal tumor therapy, enaminitrile molecular, NIR-triggered nanoparticle, domino effect, upconversion material.

## Abstract

**Rationale**: Integration of several monotherapies into a single nanosystem can produce remarkable synergistic antitumor effects compared with separate delivery of combination therapies. We developed near-infrared (NIR) light-triggered nanoparticles that induce a domino effect for multimodal tumor therapy.

**Methods**: The designed intelligent phototriggered nanoparticles (IPNs) were composed of a copper sulfide-loaded upconversion nanoparticle core, a thermosensitive and photosensitive enaminitrile molecule (EM) organogel shell loaded with anticancer drugs, and a cancer cell membrane coating. Irradiation with an NIR laser activated a domino effect beginning with photothermal generation by copper sulfide for photothermal therapy that also resulted in phase transformation of the EM gel to release the anticancer drug. Meanwhile, the NIR light energy was converted to ultraviolet light by the upconversion core to excite the EM, which generated reactive oxygen species for photodynamic therapy.

**Results**: IPNs achieved excellent antitumor effects *in vitro* and *in vivo* with little systemic toxicity, indicating that IPNs could serve as a safe and high-performance instrument for synergetic antitumor therapy.

**Conclusion**: This intelligent drug delivery system induced a chain reaction generating multiple antitumor therapies after a single stimulus.

## Introduction

Current therapies for tumor treatment include surgery, chemotherapy, photodynamic therapy (PDT), photothermal therapy (PTT), radiotherapy, immunotherapy, stem cell therapy, and hormone therapy 1,2. Integration of multiple therapeutic modalities into a single nanosystem holds great promise for synergistically improving therapeutic efficacy compared with monotherapies or combination therapies delivered separately 3-5. However, nanotechnology for multimodal tumor therapy still faces technical challenges, and synergistic therapies need to be improved. In general, multimodal therapies should be administered concurrently instead of sequentially for optimal treatment effects. Only if therapies are integrated into a nanovehicle can synergy be maximized with a single excitation source. To achieve these aims, we developed intelligent phototriggered nanoparticles (IPNs) that respond to a single stimulus and induce a domino effect for multimodal tumor therapy.

**Scheme 1:**
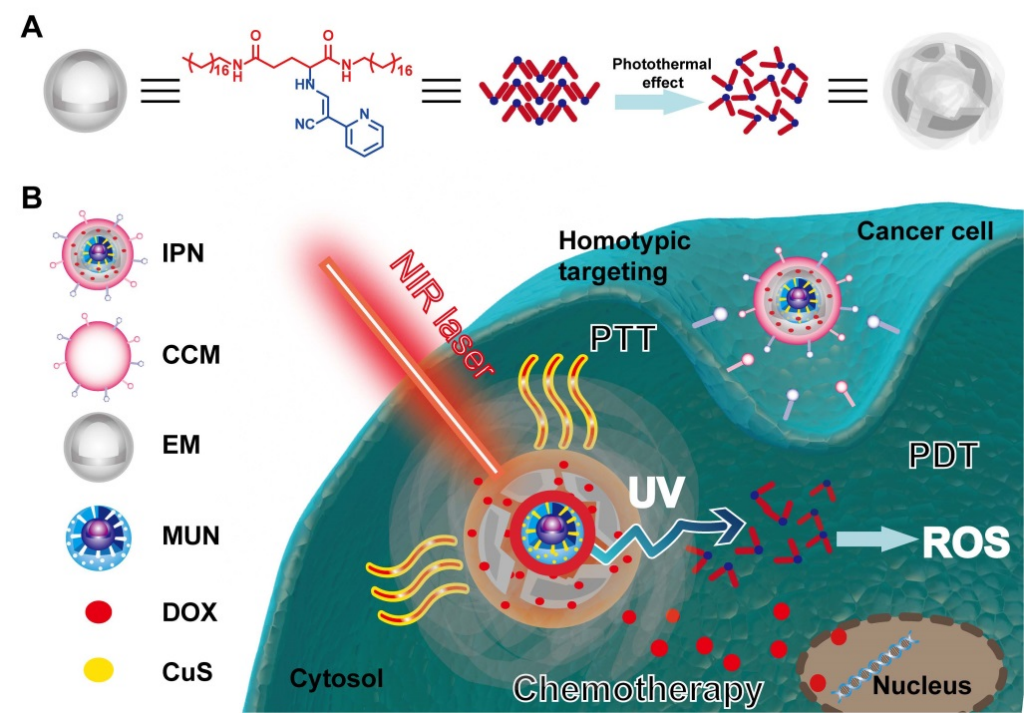
Schematic illustration of the intelligent phototriggered nanoparticles. (A) Chemical structure of EM gel and illustration of its photothermally induced phase transition. (B) Main components of IPNs and a schematic illustration of the domino effect induced by IPNs for multimodal tumor therapy.

In developing the IPNs, we first considered the specific stimulus for initiating the domino effect. Significant efforts have been devoted to the development of stimuli-responsive nanocarriers that respond to tumor-specific endogenous stimuli (e.g., pH, redox state, enzymes) and exogenous stimuli (e.g., heat, light, magnetic field, ultrasound) 6-11. Among these stimuli, light irradiation can precisely control the response at the desired time and location 12-15. Since tissues are more transparent to near-infrared (NIR) light than visible light, NIR irradiation provides deeper tissue penetration and minimal photodamage. Therefore, NIR irradiation was chosen to trigger the domino effect. Next, we considered photothermal-induced drug release and PTT as consequent responses to NIR irradiation. Compared with traditional photothermal agents (e.g., organic chemicals, carbon-based complexes, metal composites, and semiconductors) 16-19, copper sulfide (CuS) has outstanding advantages, such as low toxicity, high stability, long blood circulation, biocompatibility, and no obvious side effects from stimulation in the visible spectrum 20-22. Hence, CuS was selected as the first domino to generate heat for PTT and drug release. Thermosensitive materials that control drug release in a spatiotemporal manner have been widely applied in NIR-triggered drug delivery systems 23, 24. However, these materials still have shortcomings in tumor targeting, biodegradation, surface modification, and reversible release. Recently, Ren and coworkers reported multistimuli-responsive enaminitrile molecular (EM) switches displaying H^+^-induced aggregate emission and organogelation properties 25. EM gel possesses excellent temperature sensitivity, so we chose it as the second domino to control drug release. We also found that EM can be used as a photosensitizer in acidic environments to generate a massive amount of reactive oxygen species (ROS) with ultraviolet (UV) irradiation. However, UV light has poor tissue penetration and is phototoxic. Recently, lanthanide ion-doped upconversion nanoparticles (UCNPs) with light conversion from NIR to UV have been developed for the treatment of solid tumors 26-28. Therefore, we combined EM gel with UCNPs to enable PDT with NIR excitation. Finally, we realized the above combinations using mesoporous silica, which has a very large specific surface area to load drugs and has been widely applied as a nanocarrier 29-31. Mesoporous silica-coated UCNPs were loaded with CuS to form the nanoparticle core, which was coated with EM gel encapsulating the model antitumor drug doxorubicin (DOX). The core-shell structure of the IPNs is illustrated in Scheme 1. To enhance tumor targeting, IPNs were wrapped with cancer cell membrane (CCM), which offers unique advantages such as immune escape and homologous binding capabilities 32-34.

The designed IPNs induced a domino effect for trimodal PTT, PDT, and chemotherapy of tumors. After intravenous injection, the IPNs accumulated in tumors as a result of the enhanced permeability and retention (EPR) effect and CCM homologous binding. NIR irradiation of the tumor triggered photothermal generation by CuS, which caused PTT and resulted in phase transformation of the EM gel to release DOX. Meanwhile, the NIR light was upconverted to UV light by the UCNPs, which excited the EM to generate ROS. Without NIR irradiation, DOX encapsulation in the EM gel was stable. The amount of DOX released was controlled on demand by adjusting the irradiation time and laser on/off cycle. In summary, we developed a novel nanoplatform for multimodal therapy that achieved safe and synergistic tumor treatment *via* a domino effect.

## Methods

### Materials

DOX and the hydrophilic fluorophore Cy7 were purchased from ApexBio (Houston, USA). 3-(4,5-dimethylthiazol-2-yl)-2,5-diphenyltetrazoliumbromide (MTT) was purchased from Shanghai Yuanye Biotechnology (Shanghai, China). DAPI staining solution (5 mg/mL), MitoTracker Red CMXRos, and Annexin V-FITC/ Propidium Iodide (PI) Apoptosis Detection Kit were obtained from Yeasen Biotech (Shanghai, China). Terminal deoxynucleotidyl transferase (TdT)-mediated dUTP nick end labeling (TUNEL) apoptosis staining kit was purchased from Vazyme Biotech (Nanjing, China). Ki67 cell proliferation detection kit was purchased from Proteintech (Rosemont, USA). 2′,7′-dichlorodihydrofluorescein diacetate (DCFH-DA) and Total Antioxidant Capacity Assay Kit were purchased from Beyotime Biotechnology (Shanghai, China). Cytochrome c (cyt c) (6H2.B4) mouse monoclonal antibodies (mAb) and other antibodies were obtained from Cell Signaling Technology (Boston, USA). Breast cancer cell lines MCF-7 and 4T1 were obtained from the Cell Bank of the Shanghai Institute of Biochemistry and Cell Biology (Chinese Academy of Sciences, Shanghai, China). Dulbecco's modified Eagle medium (DMEM), Roswell Park Memorial Institute (RPMI) 1640 medium, and fetal bovine serum (FBS) were purchased from Invitrogen (Carlsbad, USA). UCnano™ mesoporous silica-coated UCNPs (NaYF_4_: Yb/Tm, UV emission) and a 980 nm laser (MDL-H-980nm-4W-18120782) were obtained from Hefei Fluonano Biotech (Hefei, China). All other chemicals used were of analytical grade, and distilled deionized water was used.

### Synthesis of EM

EM was synthesized based on the reported literature 25, 35, 36. Stearylamine (8.6 g), *N*-benzyloxycarbonyl-L-glutamic acid (4.0 g), and triethylamine (TEA, 4.4 g) were dissolved in tetrahydrofuran (THF, 400 mL). The solution was placed in an ice bath, diethylphosphorocyanidate (DEPC, 5.8 g) was added, and the mixture was stirred for 1 h. After stirring for 1 d at 25 °C, the solution was concentrated under a vacuum, and the obtained residue was dissolved in chloroform (350 mL). This solution was washed sequentially with 10% NaHCO_3_, 0.1 mol/L HCl, and distilled deionized water. Then, the solution was dried with Na_2_SO_4_, concentrated under a vacuum, and finally recrystallized from ethanol, which gave a solid white powder (*N*', *N*''-dioctyl-*N*-benzyloxycarbonyl-L-glutamide). Next, the obtained product (3.5 g) and palladium on carbon (Pd/C, 1 g) were added to 300 mL of an ethanol and THF (1:1, v/v) mixture. Then, hydrogen (H_2_) was imported into the solution for 10 h at 60 °C. Finally, the Pd/C was removed, and the solution was concentrated and recrystallized from ethanol to give a solid white powder (*N*', *N*''-dioctadecyl-L-glutamide). Triethyl orthoformate (763 μL), 2-pyridylacetonitrile (338 μL), zinc chloride (ZnCl_2_, catalytic amount), and acetic anhydride (Ac_2_O, 3.47 mL) were added to a round-bottom flask. The mixture was stirred at 120 °C under argon to obtain an oily product. This oil and *N*', *N*''-dioctadecyl-L-glutamide were added to ethanol, and the mixture was stirred at 25 °C for 1 d. The EM was purified by silica gel column chromatography.

### Electron spin resonance (ESR) analysis of EM

To detect various ROS generated by EM, ESR analysis was employed with 2,2,6,6-tetramethylpiperidine (TEMP) (for ^1^O_2_ detection) or 5,5-dimethyl-pyrroline-*N*-oxide (DMPO) (for ·OH detection). 1 mL EM (1 mg/mL) was mixed with 20 μL TEMP (1 M) or 10 μL DMPO (1 M) and then exposed to UV irradiation (365 nm, 80 mW/cm^2^, 5 min). The characteristic peak signals of ROS were detected by an ESR spectrometer (Bruker EMXplus, Germany). The settings for the ESR spectrometer were as follows: center field, 3500 G; sweep width, 200 G; microwave frequency, 9.82 GHz; modulation frequency, 100 kHz; power, 63.40 mW.

### Preparation of CCM-derived vesicles

CCM-derived vesicles were prepared based on previous reports 37, 38. MCF-7 cells were cultured in DMEM and 4T1 cells were cultured in RPMI 1640 supplemented with 10% FBS and 1% penicillin and streptomycin. MCF-7 or 4T1 cells were harvested by centrifugation at 900 ×*g* for 10 min. Then, the obtained cells were suspended in 10 mL of hypotonic lysing buffer (10 mM KCl, 20 mM Tris-HCl, and 2 mM MgCl_2_) and disrupted by homogenization. The disrupted cells were centrifuged at 4000 ×*g* for 10 min, and the supernatants were subjected to 20 passes. Then, the supernatant was centrifuged at 80,000 ×*g* for 2 h to obtain a pellet. The pellet was washed with 1 mM EDTA and 10 mM Tris-HCl to obtain CCM. CCM-derived vesicles were prepared by extruding the CCM for 10 passes through a 400 nm polycarbonate porous membrane in an extruder (Avanti Polar Lipids, Alabama, USA).

Western blotting was performed to determine the purity of the obtained CCM-derived vesicles. The vesicles were treated with 1×RIPA lysis buffer to extract total protein. Equal amounts of protein from different samples were separated by sodium dodecyl sulfate (10%) polyacrylamide gel electrophoresis. Then, the proteins on the gel were transferred to a polyvinylidene fluoride membrane and blotted with primary antibodies specific for cyt c, GAPDH, and histone H3. Secondary antibodies including anti-rabbit IgG and anti-mouse IgG were incubated with the corresponding primary antibodies. The protein samples were detected by a ChemiDOC^TM^ XRS^+^ system (BioRad Laboratories, Hercules, USA).

### Preparation of IPNs

Copper chloride (CuCl_2_, 5 mM, 5 mL) was mixed with mesoporous silica-coated UCNPs (MUNs). After stirring for 6 h, polyvinylpyrrolidone (10,000 g/mol) was added to the mixture. Then, sodium sulfide (Na_2_S) aqueous solution was added. The product, CuS-loaded MUNs (CMUNs), was centrifuged with water to improve its purity. Next, to prepare EM gel-coated CMUNs (GCMUNs), EM gel and CMUNs were mixed at a ratio of 3:1 (m/m) with heating and cooling. Afterward, the GCMUNs were mixed with DOX at various ratios. To coat CCM onto the GCMUNs, 1 mL of phosphate-buffered solution (PBS) containing 50 μg of GCMUNs was mixed with CCM-derived vesicles. The mixture was extruded 10 times through a 200 nm pore membrane in an extruder and then centrifuged at 1000 ×*g* to remove excess vesicles. The obtained IPNs were dissolved in PBS and stored at 4 ºC. GUs(Gel-coated CMUNs with CCM) were prepared with the same structure and composition as IPNs but without DOX.

### Characterization of IPNs

The morphologies of MUNs, CMUNs, GCMUNs, and IPNs were characterized by transmission electron microscopy (TEM) using an HT7700 (Hitachi High-Tech, Japan). To prepare the TEM samples, copper grids were contacted with a droplet of nanoparticles for 60 s three times, negatively stained with 1% (w/v) phosphotungstic acid for 30 s, and dried. The samples were replicated three times.

The surface charge (zeta potential, mV) of IPNs was measured by dynamic light scattering (ZEN3600, Malvern Panalytical, UK). The particle size distributions (diameter, nm) of IPNs suspended in saline, DMEM, PBS, and simulated body fluid (SBF) for 4 h and 7 d were measured by nanoparticle tracking analysis (ZetaView, Particle Metrix, Germany).

The photothermal conversion of IPNs was evaluated by irradiating IPNs (70 μg/mL of CuS in PBS, 5 mL) with an NIR laser at various power densities. The temperature of the suspension was monitored by an infrared camera (PI 400, Optris, Germany). The photothermal stability of IPNs was evaluated by irradiating IPNs suspension with 2.0 W/cm^2^ NIR light for five cycles. The photothermal conversion efficiency of IPNs was calculated by reported methods 39, 40.

### Phototriggered DOX release and cellular diffusion

Phototriggered DOX release was evaluated by irradiating IPNs (1 mg/mL of CuS in PBS) with an NIR laser (1.0, 1.5, 2.0 W/cm^2^). Released DOX in the solution was isolated by centrifugation at 12,000 rpm for 5 min and quantified using a microplate reader (SpectraMax Plus384, Molecular Devices, Sunnyvale, USA) at various timepoints.

To assess DOX diffusion in cells, MCF-7 cells were cultured with IPNs (equivalent DOX concentration of 4 μM) for 6 h. The cells were irradiated with an NIR laser (2.0 W/cm^2^, 5 min), and the nuclei were labeled with DAPI. The cells were washed with PBS three times to remove IPNs adhered to the cell surface and excess dye. Fluorescence images were taken using a high-content screening (HCS) system (ImageXpress Micro Confocal, Molecular Devices, USA).

### Cellular uptake of IPNs

Flow cytometry was employed to investigate the cellular uptake of IPNs. MCF-7 cells were seeded in 6-well plates and cultured for 24 h. The cells were incubated with fresh medium containing GCMUNs or IPNs (equivalent DOX concentration of 4 μM) for 2 h. The cells were washed, digested, and harvested by centrifugation. DOX fluorescence was detected on a flow cytometer (FACSCalibur, BD Biosciences, Franklin Lakes, USA).

### *In vitro* cytotoxicity and apoptosis induction

Live/dead cell viability assays were performed by a reported method 41. MCF-7 cells were seeded in 96-well plates and cultured for 24 h. The cells were incubated with IPNs (equivalent DOX concentration of 2 μM) and irradiated with an NIR laser (2.0 W/cm^2^, 5 min). Images of live and dead cells were captured at 12 h using an HCS system. Live cells are stained by calcein AM (green) and dead cells by ethidium homodimer-1 (red).

MTT assays were performed to assess cell proliferation. MCF-7 or 4T1 cells were seeded in 96-well plates (4.0 × 10^3^ cells per well) and cultured for 12 h. The cells were incubated with DOX, GUs, or IPNs (equivalent DOX concentrations of 0-36 μM) for 24 h. Then, the irradiation groups (GUs+L, IPNs+L) were irradiated with an NIR laser (2.0 W/cm^2^, 5 min). MTT solution was added to each well, and then 150 μL of DMSO was added to each well to dissolve the formazan. Formazan absorption was measured at 570 nm using a microplate reader. The MTT assay results were also used to calculate the combination index using CompuSyn software by the Chou-Talalay method 42.

Apoptosis induction by IPNs was assessed using an Annexin V-FITC/PI double staining kit. MCF-7 cells were seeded in 6-well plates (2 × 10^5^ cells per well) and cultured for 24 h. The cells were incubated with DOX, GUs, or IPNs (equivalent DOX concentration of 4 μM). Then, the irradiation groups (GUs+L, IPNs+L) were irradiated with an NIR laser (2.0 W/cm^2^, 5 min). After another 24 h of incubation, the cells were washed with PBS, digested by trypsin, and resuspended in 0.5 mL of annexin binding buffer. The cells were stained in binding buffer containing annexin V-FITC and PI for 15 min. The cells were then analyzed by flow cytometry.

### Intracellular ROS production

MCF-7 cells were seeded in 6-well plates (2 × 10^5^ cells per well) and cultured for 24 h. To assess ROS production by EM, the cells were incubated with 4 μM EM or 0.1% DMSO for 24 h and then irradiated with UV light (365 nm, 80 mW/cm^2^, 5 min). To assess ROS production by IPNs, the cells were incubated with DOX, GUs, or IPNs (equivalent DOX concentration of 4 μM) and then the irradiation groups (GUs+L, IPNs+L) were irradiated with an NIR laser (2.0 W/cm^2^, 5 min). ROS were reacted with DCFH-DA for 20 min at 37 °C. The relative ROS levels in the cells were measured by flow cytometry and images were captured using an HCS system.

### Intracellular cyt c distribution

A cyt c release assay was performed by immunofluorescence in MCF-7 cells. The cells were seeded in 96-well plates (1.0 × 10^4^ cells per well) and cultured for 12 h. The cells were incubated with 100 µL of IPNs (equivalent DOX concentration of 2 µM) or 0.1% DMSO for 24 h and then irradiated with an NIR laser (2.0 W/cm^2^, 5 min). Then, the cells were incubated with 1.0 μM MitoTracker, fixed in 4% paraformaldehyde for 15 min, and incubated in 5% bovine serum albumin for 1 h. The cells were then incubated with cyt c mouse mAb (1:300) overnight. Cy3-labeled goat anti-mouse IgG (1:100) was used as the secondary antibody, and DAPI was used to stain cell nuclei. The cells were visualized using an HCS system.

### Mitochondrial superoxide detection

The mesoporous silica particles were used as the core to replace MUNs to synthesize non-intelligent phototriggered nanoparticles (NIPNs). Non-upconverting NIPNs were synthesized using mesoporous silica particles in place of MUNs. The structure was confirmed by TEM. MCF-7 cells seeded in 6-well plates were incubated with 1 mL of IPNs, NIPNs, or 0.1% DMSO (equivalent DOX concentration of 4 µM) for 24 h. Then, the cells were irradiated with an NIR laser (2.0 W/cm^2^, 5 min). Mitochondrial superoxide was reacted with MitoSOX Red mitochondrial superoxide indicator (ex/em = 510/580 nm) for 20 min at 37 °C and then detected by flow cytometry.

### Tumor model generation

All animal experiments were performed in accordance with guidelines from the Animal Ethics Committee of China Pharmaceutical University and the National Institutes of Health Guide for the Care and Use of Laboratory Animals. Specified pathogen-free 5-week-old female BALB/c nude mice were purchased from Qinglongshan Animal Breeding Center (Nanjing, China). 4T1 cells (1.0 × 10^6^ cells in 100 μL of PBS) were injected into the ventral mammary fat pads of the mice.

### *In vivo* biodistribution

To evaluate the tumor targeting capability of IPNs, we replaced DOX with a fluorescent Cy7 dye to assemble CCM-labled nanoparticles (CNPs). As a comparison, nanoparticles (NPs) were also obtained based on CNPs lacking cytomembrane decoration. Mice bearing 100 mm^3^ 4T1 tumors were intravenously injected with NPs or CNPs (equivalent Cy7 dose of 5.0 mg/kg) via the tail vein. *In vivo* fluorescence imaging was performed at 1, 4, 12, and 24 h postinjection (Fusion FX7 Edge Spectra, Vilber, France). After each scan, the mice were euthanized and tissues (heart, liver, spleen, lungs, kidneys, tumors) were harvested and imaged. The fluorescence intensities of all images were analyzed using Living Image 4.0 Software.

Cu element analysis was performed following a reported method 43. Mice bearing 300-400 mm^3^ 4T1 tumors were intravenously injected with IPNs (10 mg/kg) via the tail vein. At 4, 24, 168, and 336 h postinjection, the heart, liver, spleen, lung, kidney, and tumor (n = 3) were harvested, freeze-dried, and weighed. The tissues were digested in 4 mL of aqua regia and then the solution was evaporated. The precipitate was suspended in an aqueous solution containing 1.5% HCl and 0.5% HNO_3_ and then centrifuged at 10,000 rpm for 10 min. The Cu content in the supernatant was analyzed by inductively coupled plasma mass spectrometry (ICP-MS; Elan DRC II, PerkinElmer, Waltham, USA).

### *In vivo* therapeutic efficacy and safety

To investigate the antitumor effect of IPNs *in vivo*, mice bearing 100 mm^3^ 4T1 tumors were randomly divided into seven groups (n = 6) and intravenously treated with saline, DOX, GUs, GUs+L, IPNs+L, and IPNs+L (high) once every other day for 14 days. The injected dose was equivalent to 1.0 mg/kg DOX, except for the IPNs+L (high) group, which was 2.0 mg/kg DOX. For the irradiated groups, the tumors were irradiated with an NIR laser (2.0 W/cm^2^, 15 min) 12 h postinjection. After 14 days of treatments, all mice were euthanized. The tumors and major organs (heart, liver, spleen, lungs, kidneys) were harvested and stored at -80 °C. The tissues were sectioned and stained with hematoxylin and eosin (H&E), TUNEL assay, and Ki67 with Hoechst 33342 for nuclear counterstaining. The cells were observed with an automatic multispectral imaging system (NanoZoomer Digital Pathology, Hamamatsu, Japan). To confirm intratumoral ROS generation by IPNs with NIR irradiation, the tumors were homogenized and centrifuged to extract ROS. After protein quantification, the ROS level was detected with Total Antioxidant Capacity Assay Kit using the ferric-reducing ability of plasma method.

To evaluate the safety of IPNs *in vivo*, the serum levels 44 of blood urea nitrogen (BUN), creatinine (CRE), aspartate transaminase (AST), alanine transaminase (ALT) were measured using assay kits (Lai Er Bio-Tech, China).

### Statistical analysis

The results are presented as mean ± standard deviation (s.d.). All experiments were repeated at least three times. The results were analyzed by one-way analysis of variance with Tukey multiple comparison test if appropriate (Prism 5.0, GraphPad, San Diego, USA). A *P*-value less than 0.05 was considered a significant difference. Statistical significance is indicated by *P < 0.05, **P < 0.01 and ***P < 0.001.

## Results and Discussion

### Synthesis and characterization of IPNs

**Figure 1:**
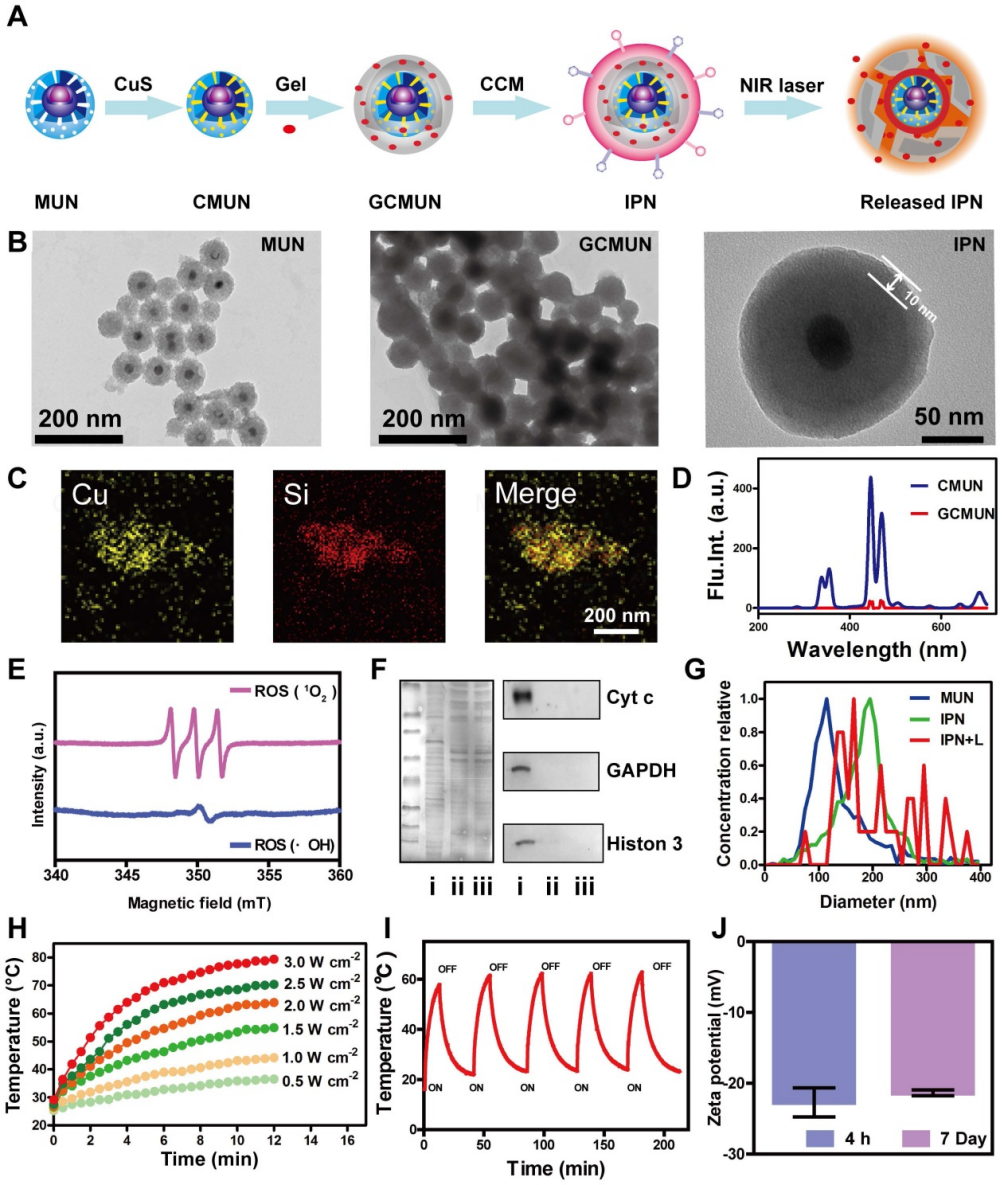
Synthesis and characterization of IPNs. (A) Schematic illustration of the formation of IPNs. (B) TEM images of MUNs, GCMUNs, and IPNs. (C) Elemental mapping images (Cu and Si) of CMUNs. (D) Fluorescence spectra of CMUNs and GCMUNs in solution with NIR excitation. (E) ESR spectra demonstrating ROS (^1^O_2_ and ·OH) generation by EM following UV irradiation for 5 min. (F) Membrane proteins by western blotting analysis in MCF-7 cell lysate (i), CCM-derived vesicles (ii), and IPNs (iii). (G) Size distribution of MUNs, IPNs, and IPNs+L in pH 7.4 buffer. (H) Temperature elevation of IPNs solution (70 μg/mL of CuS) with various NIR laser (980 nm) power densities. (I) Photothermal stability of IPNs over five on/off cycles of NIR laser irradiation. (J) Zeta potentials of IPNs at 4 h and 7 d.

Multimodal IPNs were assembled in a stepwise manner from core and shell components (Figure 1A). First, the thermosensitive and photosensitive EM shell component was synthesized (Figure S1). EM was composed of a liposoluble aliphatic chain and a glutamic acid derivative as an acid/base-activated switch. The chemical structure was confirmed by ^1^H NMR, ^13^C NMR, and MS spectroscopy (Figures S2-S4). The UV spectrum of EM showed strong absorption at 365 nm (Figure S5). Furthermore, EM emitted fluorescence at 445 nm in acidic solution (pH 4.0-5.0) but not in alkalescent solution (pH 7.4) (Figure S6). Therefore, EM displayed H^+^-induced aggregate emission. We next measured ROS generation by EM in human breast cancer MCF-7 cells. After irradiation with a 365 nm laser, ROS levels were 3.8 times higher in EM-treated cells than in the control group (Figure S7). Next, the organogelation properties of EM were studied. EM gelled in organic solvent at 120-420 mM. More interestingly, these gels disintegrated into solution above their phase transition temperatures of 45 °C (120 mM), 50 °C (150 mM), 60 °C (180 mM), and 65 °C (210 mM) (Figure S8). In summary, EM was shown to be a photosensitizer in acidic environments with excitation at 365 nm and an excellent thermosensitive agent. These unique characteristics could allow EM to be used as a vehicle for drug release and to produce a photodynamic antitumor effect in the tumor microenvironment.

Next, in order to match the UV excitation of EM, we selected biodegradable mesoporous silica-coated UCNPs (MUNs) with a diameter of 100 nm (Figure 1B). CuS, as a photothermal agent, was loaded into the pores of MUNs through a mesoporous silica *in situ* growth approach 43 to obtain CMUNs. The CuS-loading capacity was up to 9.1% based on a standard curve of Cu^2+^. This standard curve was obtained by titrating sodium diethyldithiocarbamate trihydrate, which had a linear range of 0-187.5 μM at 6 nm (Figure S9). TEM showed that CMUNs were spherical with a diameter of 110 nm (Figure S10). Element mapping images (Figure 1C) derived from energy-dispersive X-ray spectroscopy (Figure S11) further verified that the Cu and Si distribution patterns matched, signifying that CuS was uniformly distributed over the CMUNs.

Next, CMUNs were coated with the thermosensitive and photosensitive EM gel to obtain GCMUNs. GCMUNs with a diameter of 150 nm (Figure 1B) were obtained by mixing CMUNs and EM gel at an optimal mass ratio of 1:3 (Figure S12). DOX was loaded into the EM gel, which could be released through the phase-transition effect of EM to produce chemotherapy. The DOX loading efficiency was determined to be as high as 15.9% (m/m) (Figure S13). Due to the UCNP core, CMUNs emitted fluorescence at 354-445 nm with NIR (980 nm) excitation. In comparison, the fluorescence emission at 354 nm of GCMUNs was lower under the same conditions (Figure 1D). The UV light emitted by GCMUNs could be absorbed by the EM gel to produce ROS. ESR detection demonstrated that EM gel was activated by UV irradiation and generated large amounts of ROS (^1^O_2_ and ·OH) (Figure 1E). EM gel was a crucial domino causing a whole row of latter dominos to fall inducing a domino effect for multimodal tumor therapy.

Finally, GCMUNs were coated with CCM to obtain IPNs. MCF-7 cell membranes were prepared through a combined extraction method 36. TEM demonstrated that the prepared CCM-derived vesicles had a particle size of ~180 nm (Figure S14). To assess retention of membrane proteins on the CCM-derived vesicles and IPNs, gel electrophoresis and western blotting were performed. As shown in Figure 1F, the protein profile of IPNs closely matched that of the CCM-derived vesicles rather than MCF-7 cell lysate. Moreover, protein markers for the nucleus (histone 3), mitochondria (cyt c), and cytosol (GAPDH) were present in low quantities on the CCM-derived vesicles and IPNs, indicating good purification and preservation of membrane proteins. A clear TEM image of IPNs shows that the thickness of the CCM coating was ~10 nm (Figure 1B), and IPNs had a particle size of ~190 nm (Figure 1G). Element mapping images of IPNs visualizing all the components of the final nanoparticles are shown in Figure S15. The UV-vis-NIR absorption spectra and fluorescence emission spectra (ex = 980 nm) of MUNs and IPNs were collected to confirm their optical properties (Figures S16, S17). At last, the photothermal effect of IPNs was studied. Irradiation of IPNs solution with an NIR laser (980 nm) raised the solution temperature to 36.4-78.1 °C with various power densities (Figures 1H, S18). As shown in Figure 1I, IPNs demonstrated stable photothermal performance for at least five cycles of NIR laser irradiation. According to the linear regression curve of the cooling stage versus the negative natural logarithm of driving force temperature (Figure S19), the photothermal conversion efficiency of IPNs was calculated to be 61.4%. Moreover, IPNs were stable in saline, DMEM, PBS, and SBF; no increase in surface charge (Figure 1J) or particle size (Figures S20, S21) was observed over 7 days. There was also no obvious change in the UV absorption spectrum of IPNs in 100% FBS after 7 days (Figure S22).

### *In vitro* drug release and phototherapy with IPNs

**Figure 2:**
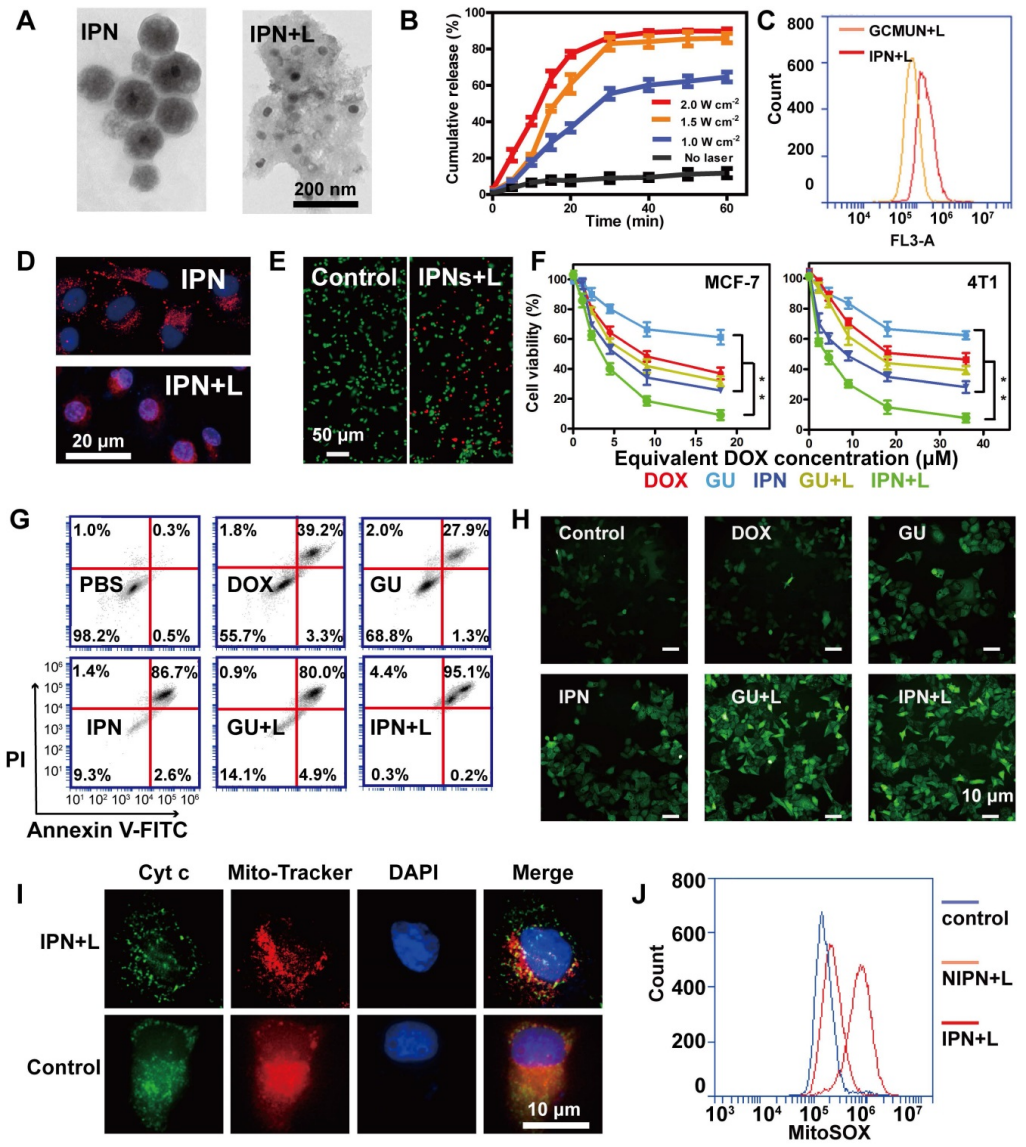
NIR light responses of IPNs. (A) TEM images of IPNs before and after NIR laser irradiation. (B) *In vitro* DOX release from IPNs triggered by NIR laser irradiation (n = 3). (C) Flow cytometry analysis of DOX fluorescence intensity in MCF-7 cells from GCMUNs and IPNs. (D) Confocal fluorescence microscopy images showing DOX (red) entrance into the nucleus (blue) before and after NIR laser irradiation. (E) Live/dead cell staining. Scale bar: 50 μm. (F) MTT cytotoxicity assay of MCF-7 and 4T1 cells treated with various formulations after 24 h of incubation (n = 3). **P < 0.01. (G) Annexin V-FITC/PI apoptosis detection of MCF-7 cells treated with various formulations after 24 h of incubation. (H) DCFH-DA detection of intracellular ROS in MCF-7 cells treated with various formulations. Scale bar: 10 μm. (I) Immunofluorescence analysis of the intracellular distribution of cyt c. Scale bar: 10 μm. (J) Mitochondrial superoxide levels in control, NIPNs+L and IPNs+L groups.

Complete deconstruction of IPNs was observed upon NIR laser irradiation, whereupon the particle size expanded from 190 nm to 90-400 nm (Figures 1G, 2A). This obvious result from heating further validated the photothermal efficiency of IPNs. Additionally, this photothermally induced deconstruction should release DOX from IPNs. Thus, DOX release from IPNs under NIR laser irradiation was evaluated in MCF-7 cells (Figure 2B). IPNs exhibited low DOX release (11.7%) over 60 min incubation without irradiation. By contrast, IPNs showed rapid DOX release when irradiated at 1.0 W/cm^2^ (64.6%), 1.5 W/cm^2^ (86.0%), and 2.0 W/cm^2^ (89.8%), confirming NIR-triggered photothermally induced drug release from IPNs. Cell uptake of nanoparticles is a crucial characterization to assess their potential bioavailability. The CCM coating helped IPNs enter MCF-7 cells more quickly than GCMUNs, resulting in increased cell uptake (Figure 2C). We also monitored DOX release and distribution in cells using an HCS system (Figure 2D). Without NIR laser irradiation, DOX fluorescence (red) was punctate because the IPNs remained stable in the cytoplasm. After irradiation, DOX fluorescence in the cytoplasm was diffuse, and violet fluorescence, indicating colocalization of DOX and the nucleus (blue), gradually increased, suggesting that DOX was released from IPNs and entered the cell nucleus. These results demonstrated that IPNs rapidly generated heat under NIR laser irradiation in cells, which destroyed the EM gel and promoted DOX release.

Next, we investigated the cytotoxicity of IPNs in human (MCF-7) and mouse (4T1) breast cancer cells. GUs were assembled as IPNs without DOX loading, and two NIR laser irradiation groups (IPNs+L and GUs+L) were used to contrast the antitumor effects of IPNs. A live/dead cell assay demonstrated the toxicity of IPNs with irradiation according to the appearance of large numbers of dead cells (red), and the morphology of cells changed (Figure 2E, S23). An MTT assay showed that all groups exhibited dose-dependent cytotoxicity to MCF-7 and 4T1 cells (Figure 2F). 38.8% of MCF-7 cells were killed by GUs, while 68.2% of MCF-7 cells were killed in the GUs+L group owing to the photothermal and photodynamic effects. With the addition of DOX, 90.9% of MCF-7 cells were killed by IPNs+L, which was due to the synergistic effect of PTT, PDT, and chemotherapy. Similar results were found in the 4T1 cells. For combination cancer therapies, the combination index (CI) is generally utilized to calculate synergy. CI values of <1, 1, and >1 represent synergistic, additive, and antagonistic effects, respectively. CI values of 0.3-0.6 were calculated for IPNs in 4T1 cells (Figure S24), suggesting synergistic effects from the chemo-photodynamic therapy combination. Apoptosis induction was also assessed in MCF-7 cells using Annexin V-FITC and PI (Figure 2G). IPNs+L induced a higher apoptosis rate (95.3%) than IPNs (89.3%), GUs (29.2%), GUs+L (84.9%), and DOX (42.5%).

ROS production during PDT results in cell death through apoptotic mechanisms. Therefore, intracellular ROS production was assessed using the cell-permeable fluorophore DCFH-DA. Flow cytometry results showed that ROS levels in MCF-7 cells treated with IPNs+L and GUs+L were 2.7 and 2.8 times higher than those in cells treated with IPNs and GUs, respectively (Figures 2H, S25). These data confirmed that the EM gel coating in IPNs and GUs acted as an NIR photosensitizer. Release of cyt c from mitochondria to cytosol is a major caspase activation pathway, often defining the point of no return in apoptosis 45, 46. Cyt c translocation stimulated by excess ROS was investigated by immunofluorescence. As shown in Figure 2I, cyt c (green) in the control group was located in the mitochondria (red), resulting in orange colocalization signal. In comparison, orange signal in the mitochondria was lower and green fluorescence appeared in the cytosol in the IPNs+L group, indicating translocation of cyt c from the mitochondria into the cytosol. Mitochondria and cyt c colocalization was calculated by Pearson's correlation coefficient (PCC) method (Figure S26). Colocalization in the IPNs group (PCC = 0.009) was lower than that in the control group (PCC = 0.942). Therefore, these results further confirmed that IPNs are efficient agents for synergistic PTT, PDT, and chemotherapy. Above all, we demonstrated the single-track antitumor process of the domino effect. To more rigorously demonstrate the domino effect that results in PDT, we removed the UCNPs from IPNs to generate NIPNs (non-intelligent phototriggered nanoparticles). We dismantled one of the dominoes to attempt to interrupt the domino effect. Increased ROS in mitochondria is a major indicator of the mitochondria-mediated apoptosis pathway. Therefore, we used the indicator MitoSOX to verify whether the apoptotic pathway is activated in the absence of UCNPs. Flow cytometry results showed that MitoSOX levels in MCF-7 cells treated with NIPNs+L and IPNs+L were 1.3 and 4.3 times higher than those in the control group, respectively (Figures 2J, S27). Therefore, the domino effect resulting in PDT was interrupted in NIPNs. These results collectively confirmed that IPNs are efficient agents for multimodal tumor therapy.

### *In vivo* biodistribution of IPNs

**Figure 3:**
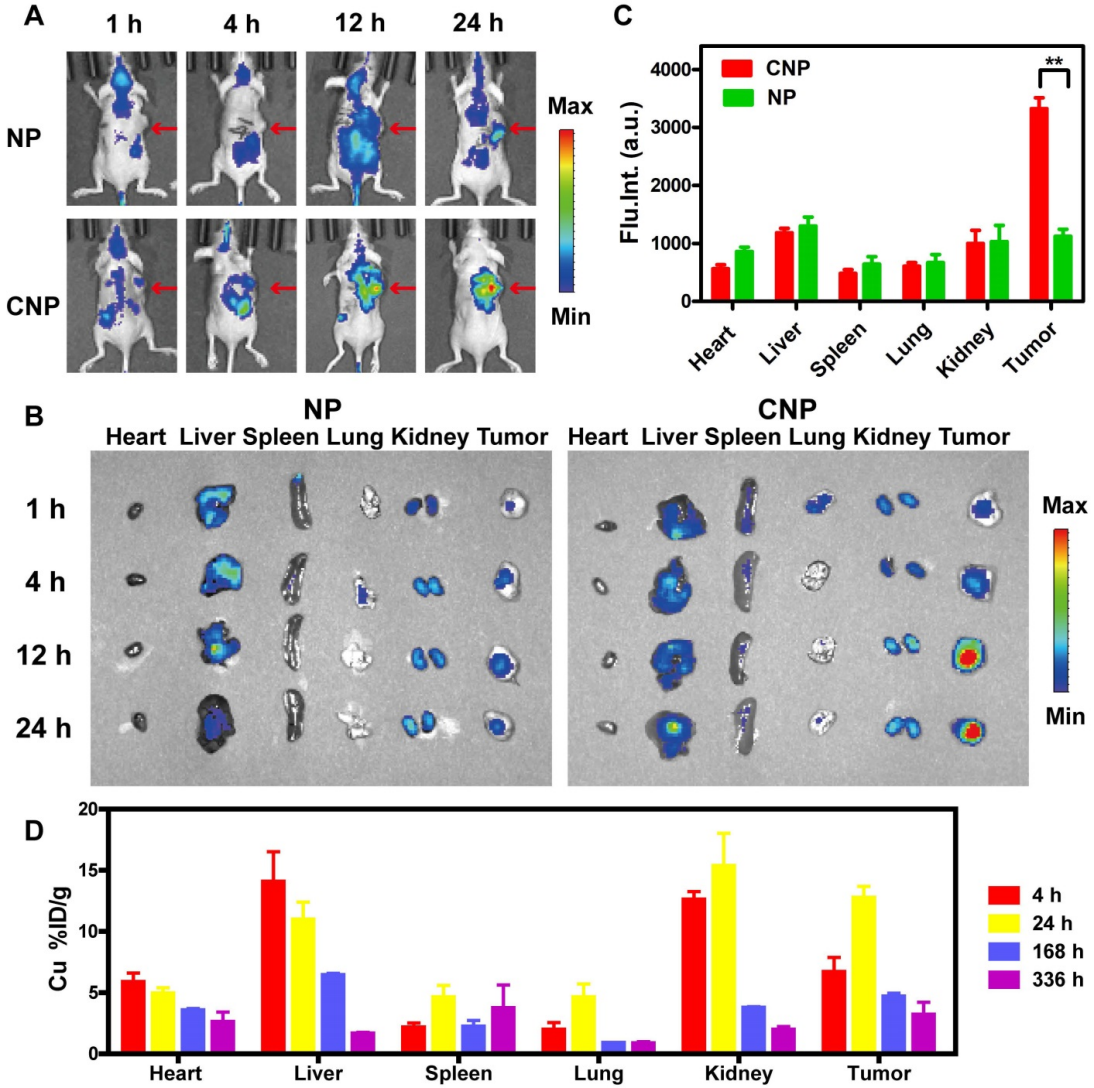
*In vivo* biodistribution of IPNs. (A) *In vivo* fluorescence images of 4T1 tumor-bearing nude mice 1, 4, 12, and 24 h after intravenous injection of NPs or CNPs at a Cy7 dose of 5.0 mg/kg. Arrows indicate tumor regions. (B) *Ex vivo* fluorescence images of tumors and other tissues harvested at 1, 4, 12 and 24 h postinjection. (C) Region-of-interest analysis of fluorescence intensity in visceral organs and tumors collected at 24 h postinjection (n = 3). **P < 0.01. (D) Distribution of Cu in tumor and main organs 4, 24, 168, and 336 h after intravenous injection of IPNs (n = 3).

IPNs could possess homotypic targeting capacity due to their CCM coating in addition to passive tumor targeting due to the EPR effect. To evaluate the tumor targeting capability of IPNs, we replaced DOX with Cy7 to assemble nanoparticles with (CNPs) and without (NPs) CCM coating. Then, the *in vivo* biodistributions of NPs and CNPs in a 4T1 xenograft mouse model after intravenous injection were studied by noninvasive NIR fluorescence imaging. Compared with NPs, CNPs showed stronger Cy7 signal in the tumor region at early timepoints (Figure 3A). Cy7 signal from CNPs in the tumor region also gradually increased over time compared with normal tissue. The signal-to-noise ratio (S/N) in the tumor was calculated from the *in vivo* images (Figure S28). The S/N value of NPs was 1.3 at 1 h and slightly increased to 3.7 at 24 h, while the S/N value of CNPs continuously increased to 8.1 at 24 h. At each indicated timepoint, some of the mice were euthanized and their heart, liver, spleen, lung, kidney, and tumor tissues were harvested for *ex vivo* imaging. The Cy7 signal from CNPs in tumor tissue was significantly higher than that of NPs at 12 h and 24 h (Figure 3B). The fluorescence intensity from CNPs in tumors was 3.0 times higher than that of NPs at 24 h, as determined by semi-quantitative region-of-interest analysis (Figure 3C). These *ex vivo* results further demonstrated the tumor-targeting capacity of IPNs. These imaging results were confirmed by quantitative ICP-MS analysis of Cu from IPNs in major organs and tumors at 4, 24, 168, and 336 h postinjection. As shown in Figure 3D, IPNs had a decent tumor uptake of 12.7%ID/g at 24 h (n = 3). Despite the high level of Cu in the liver, Cu remained in the kidneys at 4 h, indicating that IPNs might be excreted renally.

### *In vivo* therapeutic efficacy and safety of IPNs

**Figure 4:**
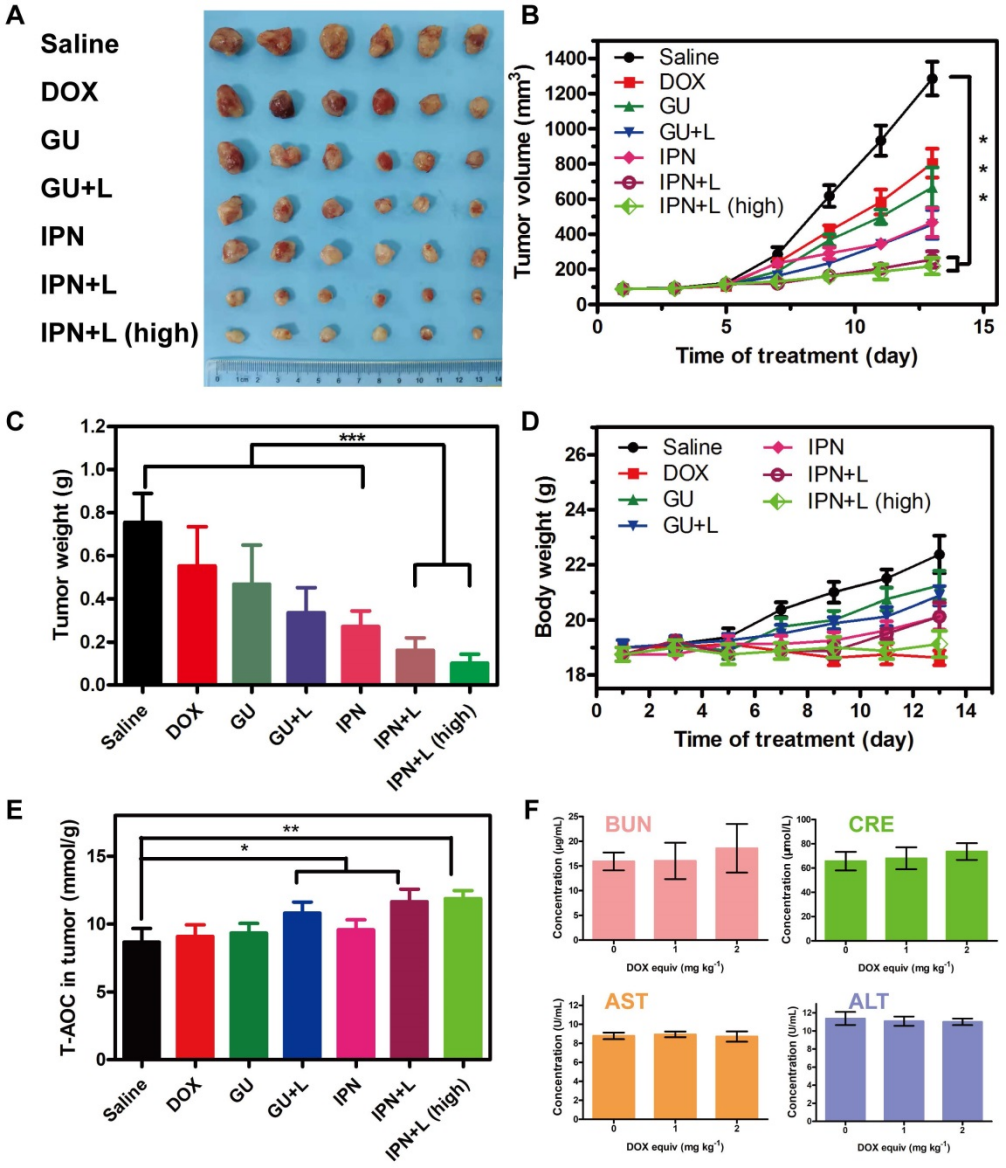
*In vivo* therapeutic efficacy and safety of IPNs. (A) Photographs of tumor tissues excised on day 14 following various treatments. (B) Tumor growth curves over 14 days during various treatments (n = 6). ***P < 0.001. (C) Weights of tumor tissues excised on day 14 following various treatments (n = 6). ***P < 0.001. (D) Body weight changes in tumor-bearing mice during various treatments (n = 6). (E) Total antioxygen capacity in tumors after 14 days of various treatments. (F) Blood biochemistry tests of BUN, CRE, GOT, and GPT after intravenous injection of various doses of IPNs (n = 3).

The *in vivo* anti-tumor therapeutic effects of IPNs were tested in 4T1 xenograft mice. Seven groups were treated: saline, DOX, GUs, GUs+L, IPNs, IPNs+L, and IPNs+L (high) (Figure S29). Compared with the saline group, DOX alone showed unsatisfactory tumor inhibition, while both IPNs+L and IPNs+L (high) presented remarkably higher inhibition of tumor growth (Figure 4A-C). The tumor inhibition rates of IPNs+L and IPNs+L (high) were 79.6% and 82.8%, respectively, at 14 days of treatment (Figure S30). More importantly, an obvious difference in tumor inhibition between non-irradiated and irradiated groups (GUs vs. GUs+L, IPNs vs. IPNs+L) was observed (Figures 4B, S30), suggesting the significant role of NIR-triggered antitumor activity. The irradiation control group showed a slight antitumor effect, similar to that of the GUs group (Figures S31-S33). The total antioxygen capacity levels of tumor cells treated with DOX, GUs, GUs+L, IPNs, IPNs+L, and IPNs+L (high) were 1.04, 1.07, 1.24, 1.10, 1.34, and 1.37 times higher than those of the control group, respectively (Figures 4E, S34). Intratumoral ROS generation in the IPNs+L group was also significantly higher than that of the control group (**P < 0.01). The dose-dependent toxicity of IPNs was investigated by liver and kidney function indexes (Figures 4F, S35). Liver function markers GOT and GPT and kidney function markers BUN and CRE were determined to be normal and not significantly different from those of the control group, suggesting that IPNs did not induce obvious hepatic or kidney disorders.

**Figure 5:**
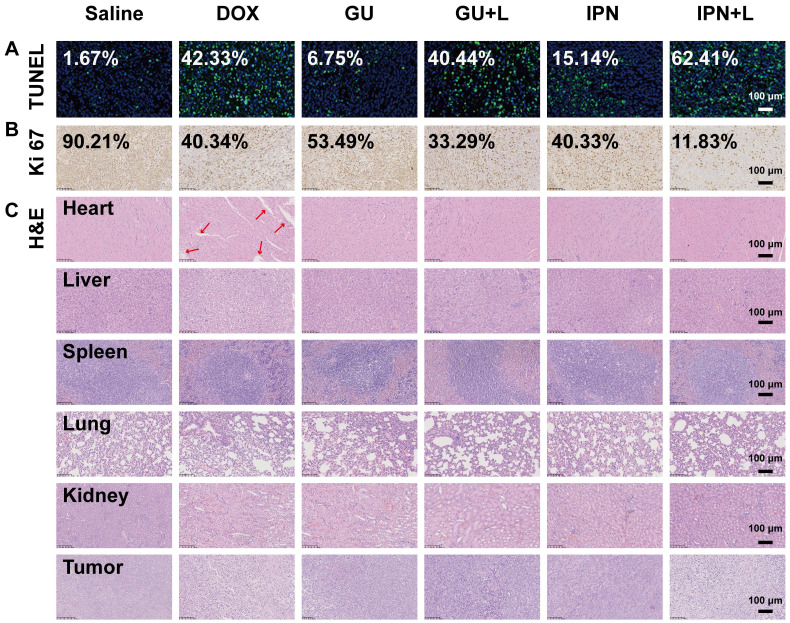
Histological effects of IPNs. (A) Detection of apoptosis in tumor tissues by TUNEL assay (green). Cell nuclei were counterstained with Hoechst (blue). (B) Images of tumor tissues stained by Ki67 immunohistochemistry. Nuclei were stained blue, and Ki67-positive cells were stained brown. (C) Histological observation of tumor tissues and major organs by H&E.

An *in situ* TUNEL assay (green) was applied to detect apoptotic cells in tumors. IPNs+L (high), IPNs+L, IPNs, GUs+L, GUs, and DOX induced, respectively, 52.6, 37.4, 9.1, 24.2, 4.0, and 25.4 times higher tumor cell apoptosis than control (Figure 5A). Ki67 mAb was applied to detect cell proliferation in tumor tissues. Ki67 staining of tumor cells in the IPNs+L (high), IPNs+L, IPNs, GUs+L, GUs, and DOX groups were 0.08, 0.13, 0.45, 0.37, 0.59, and 0.45 times that of the control group (Figure 5B). H&E staining was further used to analyze the tumor tissues by histology. Compared with the other treatment groups, the IPNs+L group exhibited massive cancer cell remission and the highest tumor cell death (Figure 5C). These results demonstrated that NIR-triggered IPNs generated distinct anti-tumor therapeutic effects *in vivo* by inducing cell apoptosis and inhibiting cell proliferation.

In addition to their therapeutic effects, we also evaluated the safety of IPNs. The body weights of mice in the IPNs+L (high), IPNs+L, IPNs, GUs+L, GUs, and saline groups slightly increased or did not significantly change during treatment, while DOX caused significant loss of body weight (Figure 4D). Furthermore, H&E staining showed that DOX caused typical cardiotoxicity indicated by cardiomyocyte vacuolation (Figure 5C). In contrast, few histological abnormalities in major organs (heart, liver, spleen, lung, and kidney) were found in the IPNs+L (high), IPNs+L, IPNs, GUs+L, and GUs groups due to the targeted delivery to the tumor, indicating that IPNs have a satisfactory safety profile for combination antitumor therapy.

## Conclusions

In summary, we successfully fabricated an intelligent phototriggered nanoplatform that induces a domino effect for multimodal tumor therapy. The designed IPNs were composed of a CuS-loaded UCNP core, thermosensitive and photosensitive EM gel shell loaded with DOX, and a CCM coating. Upon NIR irradiation of IPNs, the CuS generated heat, which not only caused PTT but also resulted in phase transformation of the EM gel to release DOX for chemotherapy. Meanwhile, the NIR light energy was converted to UV light by the UCNP core, which excited EM to generate ROS for PDT, circumventing the limited tissue penetration of UV light. Therefore, IPNs were activated by NIR light and produced trimodal PTT, PDT, and chemotherapy for synergistic tumor treatment. Furthermore, IPNs achieved excellent antitumor efficacy *in vitro* and *in vivo* with few side effects. These results indicate that IPNs could serve as a safe and high-performance instrument for multimodal tumor therapy with a single stimulus.

## Supplementary Material

Supplementary figures.
