# Supplementary material for: Intelligent phototriggered nanoparticles induce a domino effect for multimodal tumor therapy
Source: Theranostics. 2021 Apr 19;11(13):6477–90. doi: 10.7150/thno.55708 (PMC8120229; doi:10.7150/thno.55708)
Supplement: Supplementary file 1 — Supplementary figures. [file thnov11p6477s1.pdf]

Supplemental Information

Intelligent phototriggered nanoparticles induce a domino effect for multimodal tumor therapy

Xiao Xu[a,1], Chao Han[a,1], Can Zhang[b], Dan Yan[a], Chunling Ren[a], Lingyi Kong[a,*]

[a]*State Key Laboratory of Natural Medicines, Jiangsu Key Laboratory of Bioactive Natural Product Research, School of Traditional Chinese Pharmacy, China Pharmaceutical University, 24 Tong Jia Xiang, Nanjing 210009, PR China.*

[b]*State Key Laboratory of Natural Medicines, Center of Drug Discovery and Department of Pharmaceutics, China Pharmaceutical University, 24 Tong Jia Xiang, Nanjing 210009, PR China.*

[*]Corresponding author：

Lingyi Kong

E-mail address: cpu_lykong@126.com

Tel./Fax: +86 25 8327 1405.

[1]The authors contributed equally to this work.

Supplemental Figures

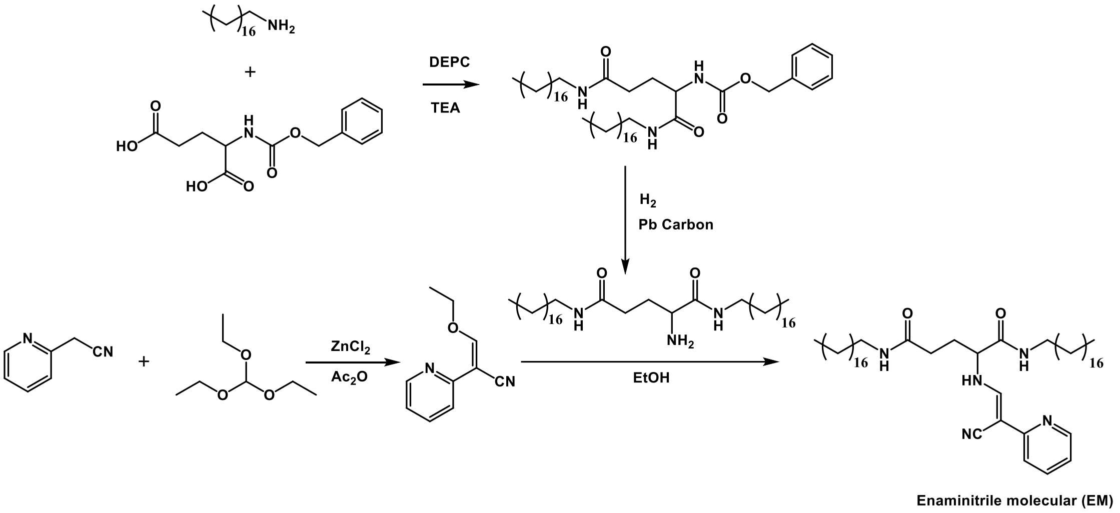

Figure S1. Synthetic route of of EM ((S,E)-2-((2-cyano-2-(pyridin-2-yl)vinyl)amino) -N1,N5-ioctadecylpentanediamide).

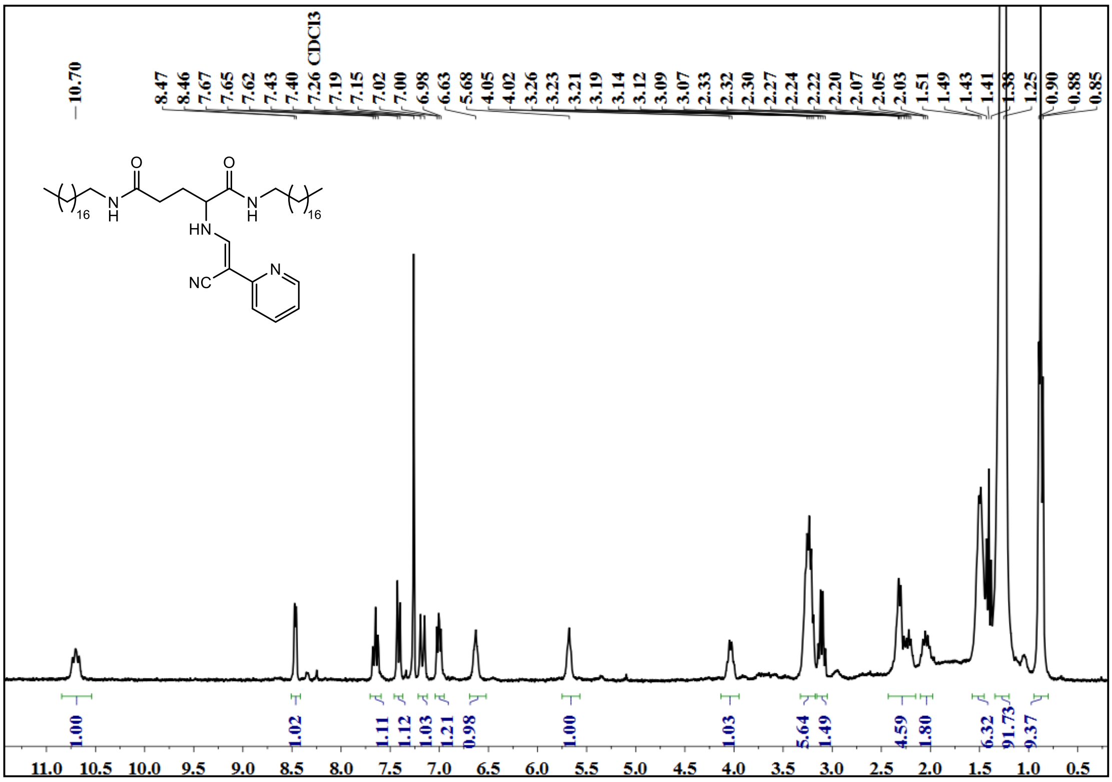

Figure S2. ^{1}H NMR spectrum of EM (300 MHz, $CDCl_3$).

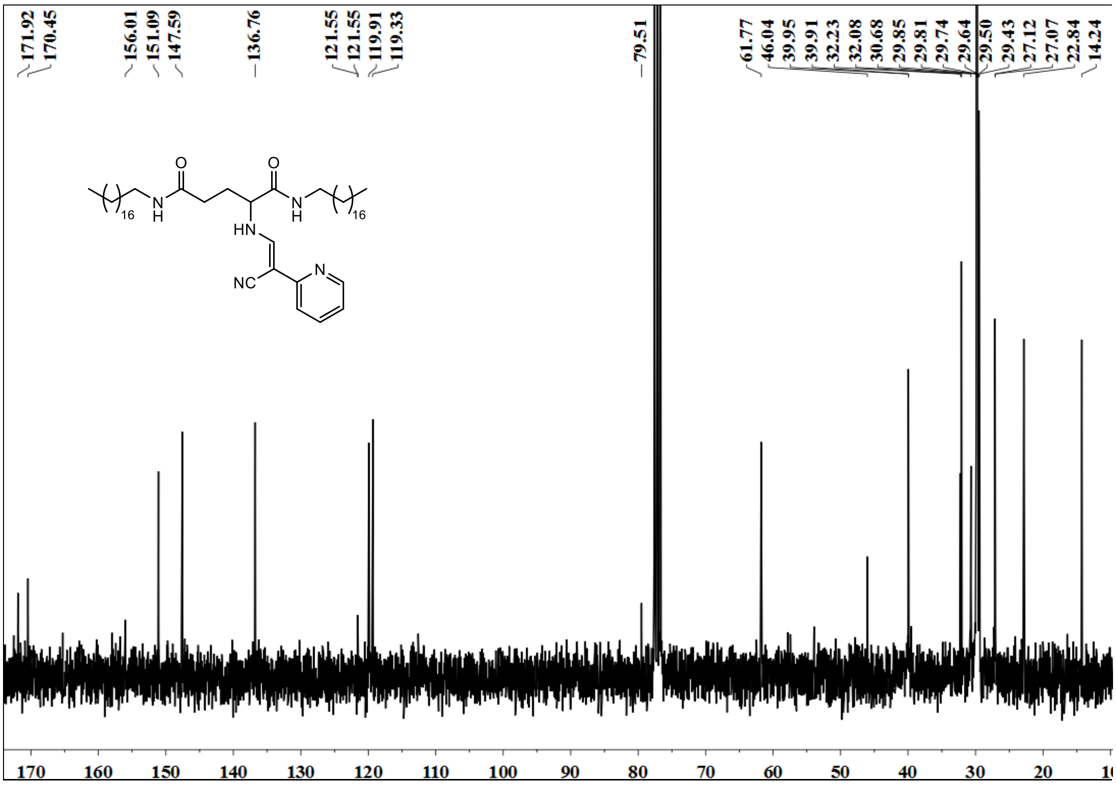

Figure S3. ^{13}C NMR spectrum of EM (75 MHz, $CDCl_3$).

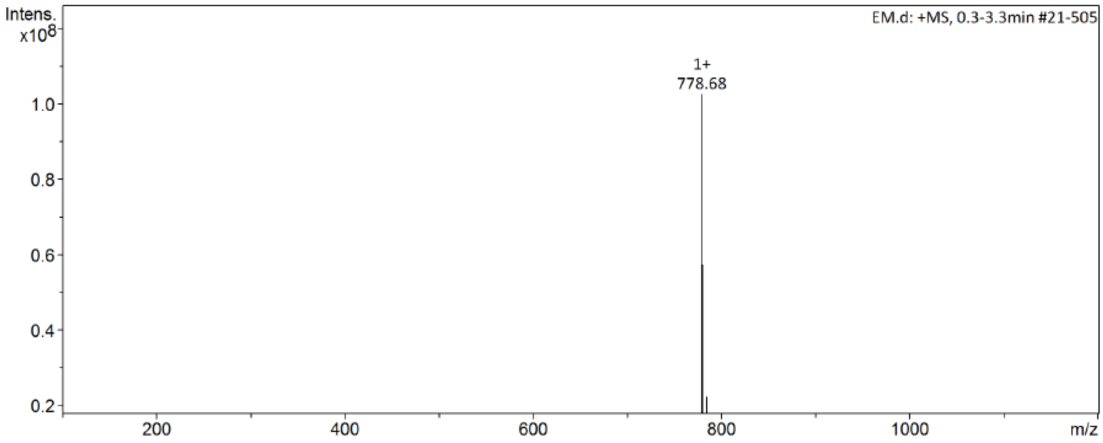

Figure S4. Mass spectrum of EM.

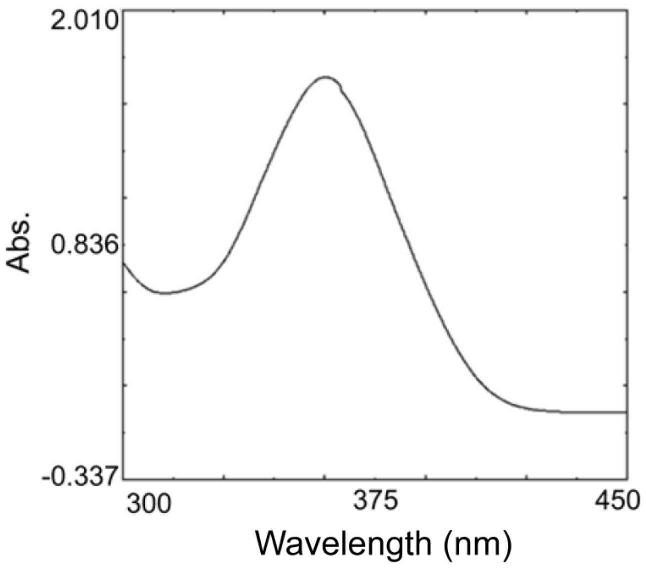

Figure S5. UV–vis absorption spectrum of EM.

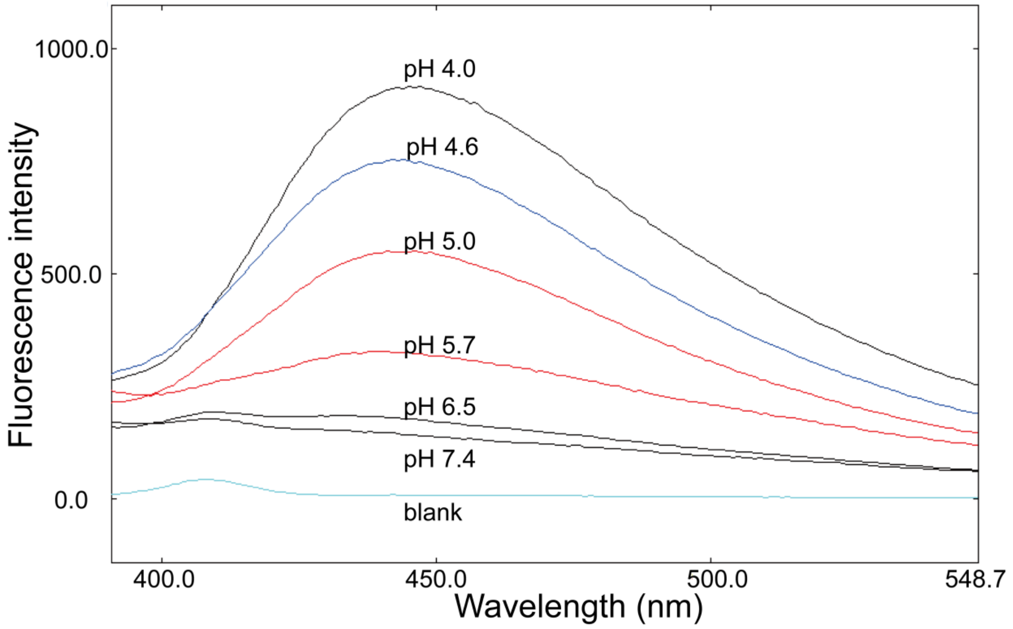

Figure S6. Fluorescence spectra of EM (0.5 mM EM in PBS containing 0.1% DMSO).

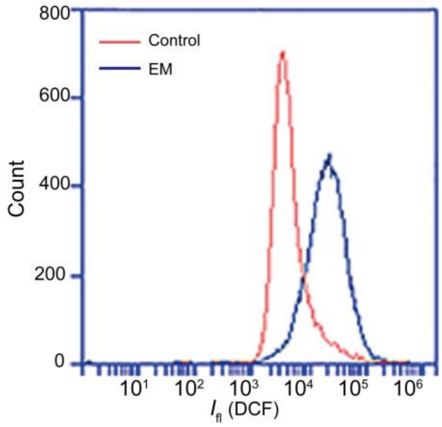

Figure S7. Intracellular ROS production treated with EM after UV light irradiation in MCF-7 cells.

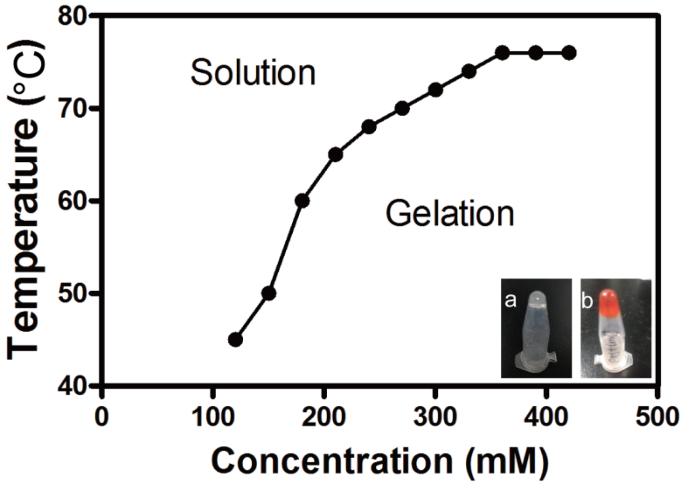

Figure S8. The temperature phase diagram of EM gel. The inset shows the photo of EM gel (a) and EM gel with DOX (b).

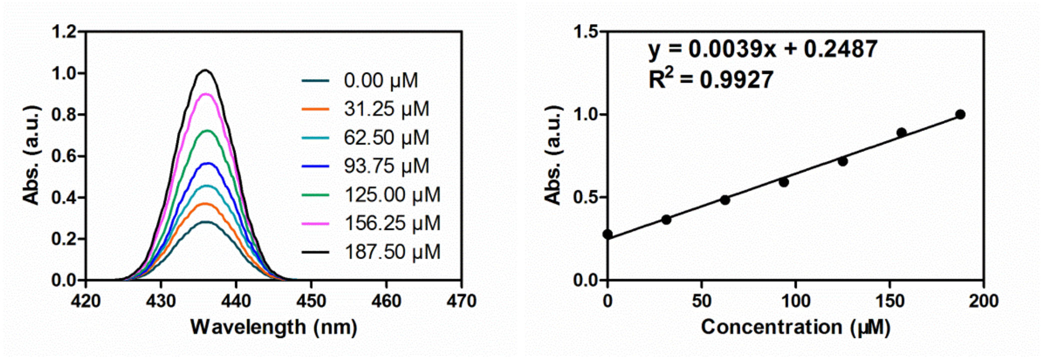

Figure S9. UV–vis spectra change of Cu(II) ion in PBS buffer and the linear correlation between the intensity (436 nm) and the Cu(II) concentration.

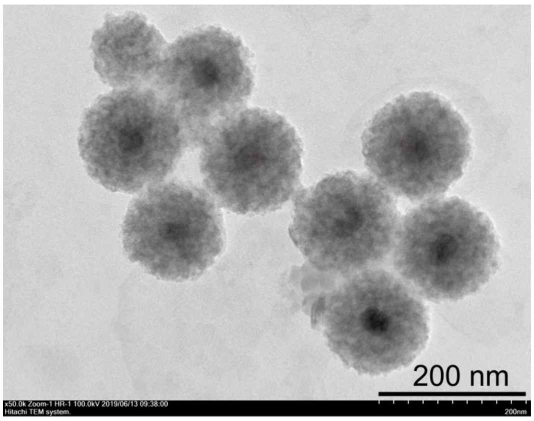

Figure S10. TEM image of CMUNs.

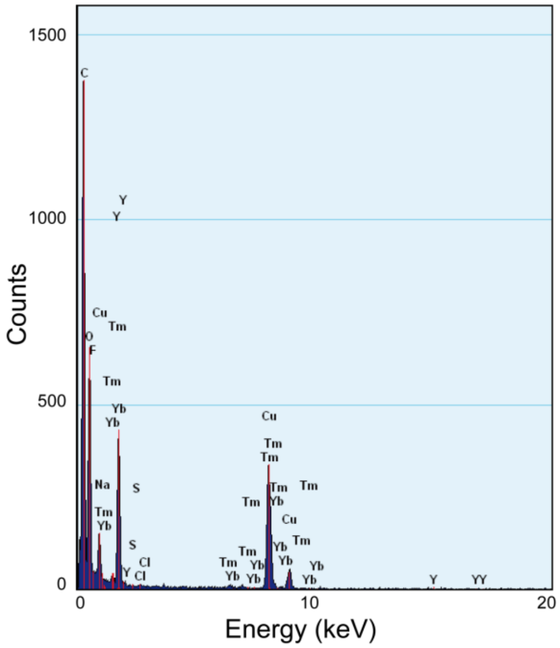

Figure S11. EDX element analysis of CMUNs.

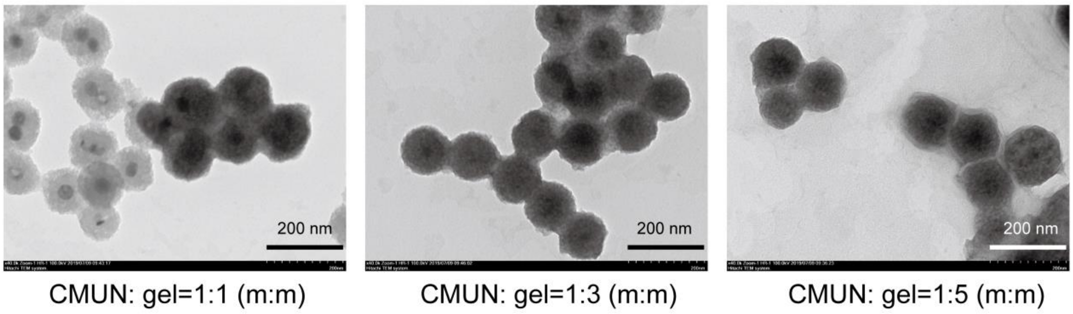

Figure S12. TEM images of GCMUNs with different quantities of CMUNs and EM gel.

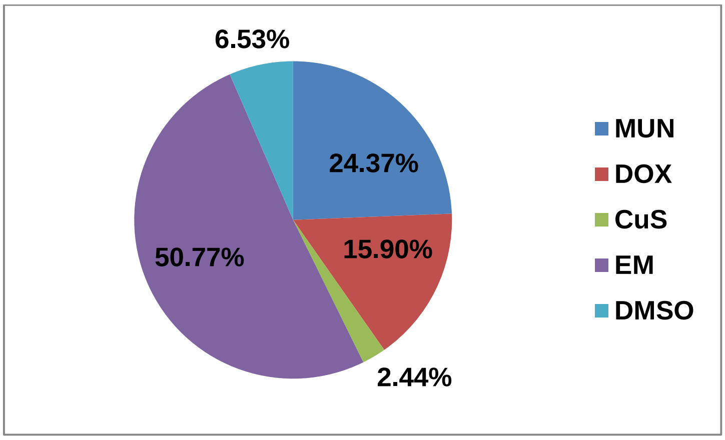

Figure S13. The weight percentage of constituent components in GCMUN.

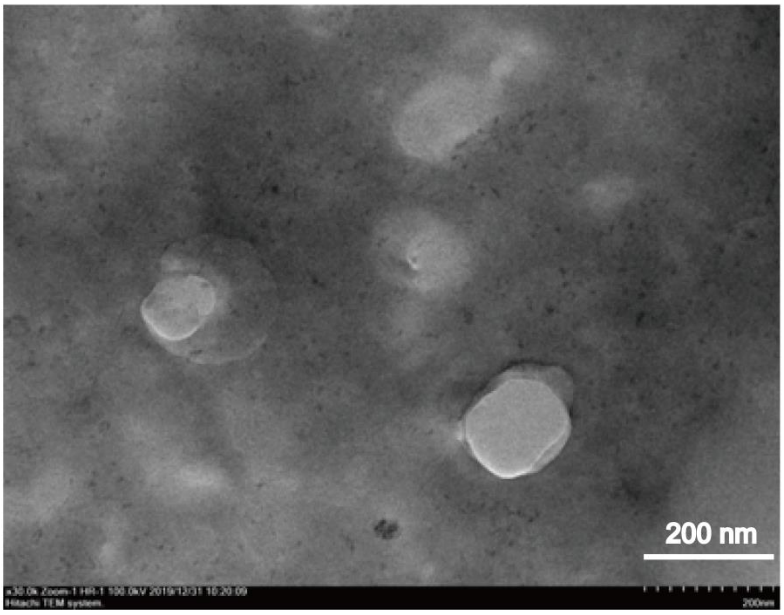

Figure S14. TEM image of the CCM-derived vesicles.

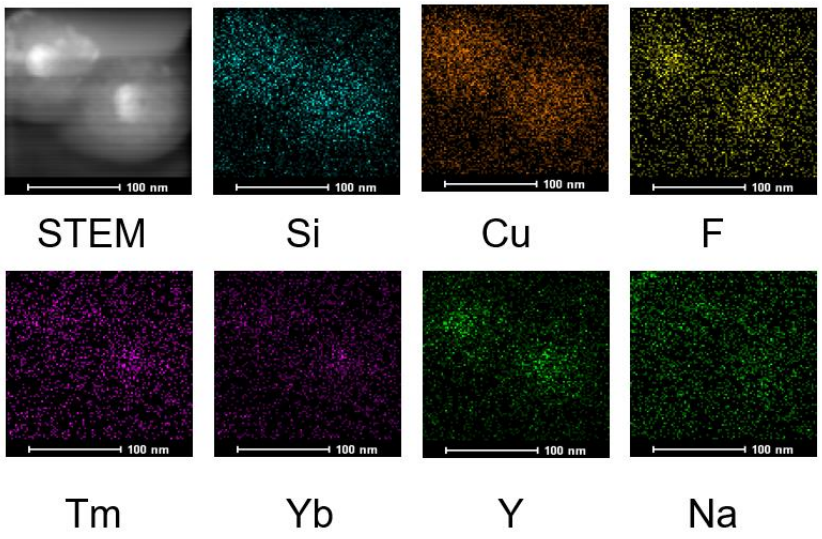

Figure S15. Elemental mapping images (Si, Cu, F, Tm, Yb, Y, and Na) of IPNs.

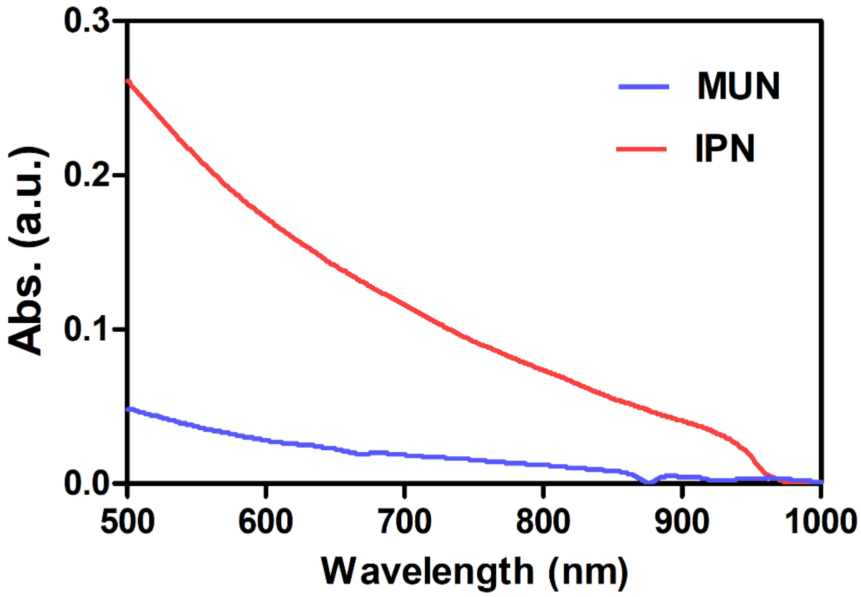

Figure S16. UV-vis-NIR absorbance spectra of MUNs and IPNs by PerkinElmer Lambda 750 UV-vis-NIR spectrophotometer.

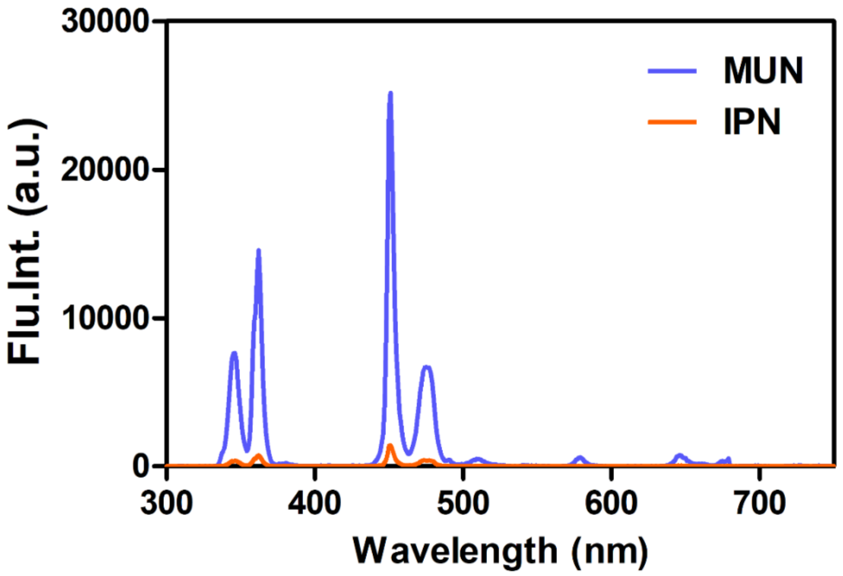

Figure S17. The fluorescence emission spectra (Ex = 980 nm) of MUNs and IPNs.

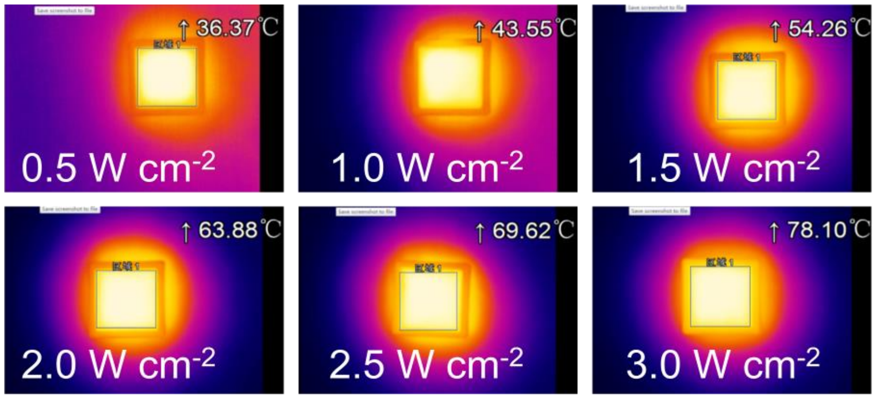

Figure S18. Thermal images of IPNs solution after different NIR laser power (980 nm) irradiation at 12 min.

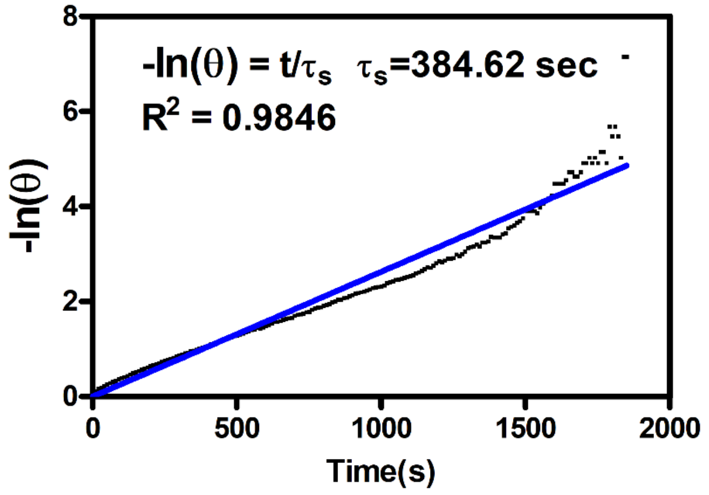

Figure S19. The linear regression curve between cooling stage and negative natural logarithm of driving force temperature of IPNs.

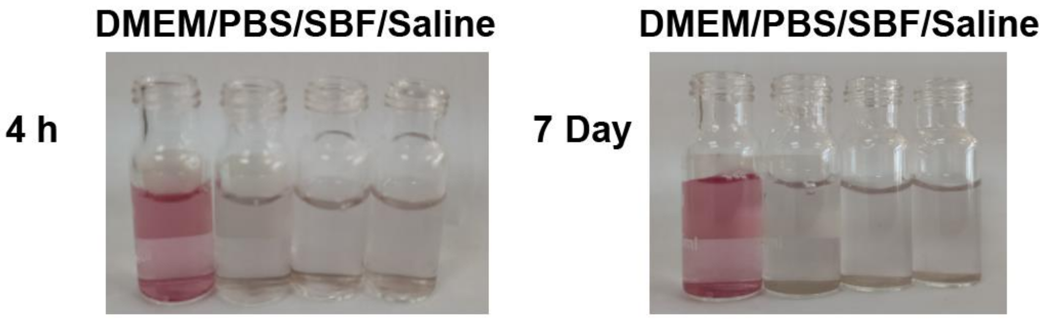

Figure S20. The images of IPNs in saline, DMEM, PBS, and SBF at 4 h and 7 day.

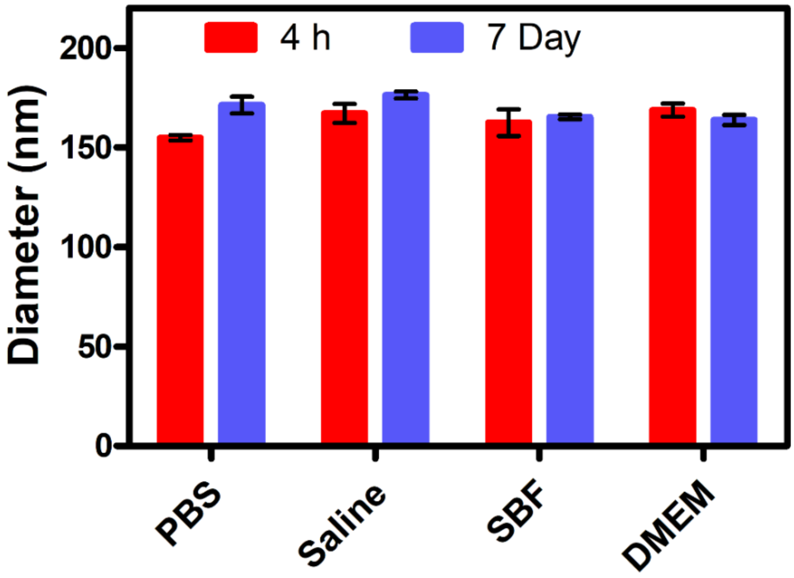

Figure S21. The particle size of IPNs in saline, DMEM, PBS, and SBF at 4 h and 7 day.

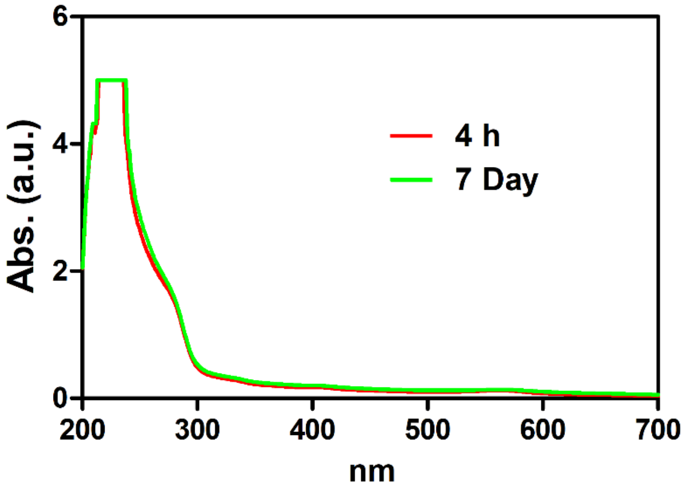

Figure S22. UV absorption spectra of IPNs in 100% fetal bovine serum at 4 h and 7 day.

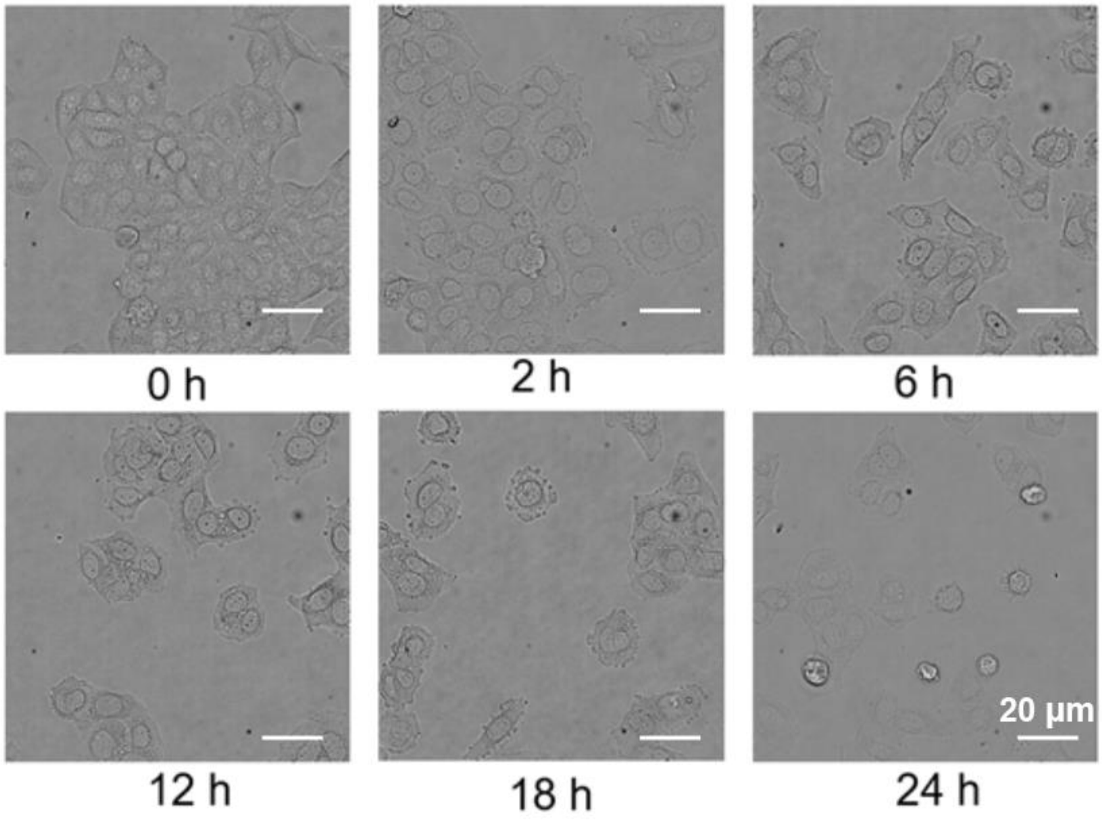

Figure S23. Morphologies of cells analyzed by bright-field microscopy. Scale bar: 20 μm.

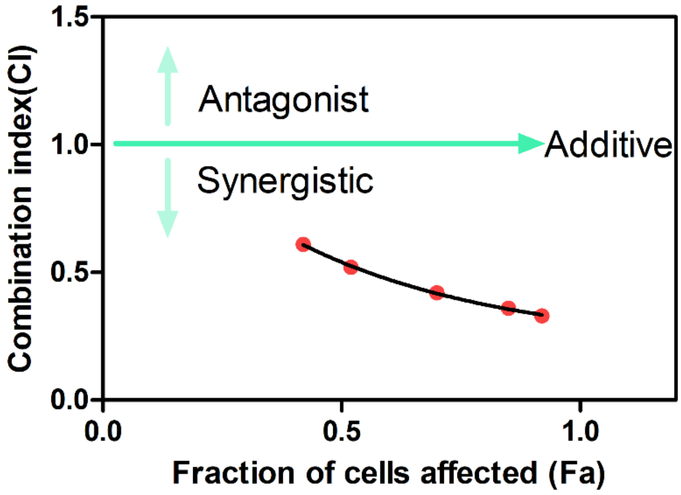

Figure S24. The combination index (CI)-plot of 4T1 cells treated with IPNs mediated chemo-photodynamic therapy.

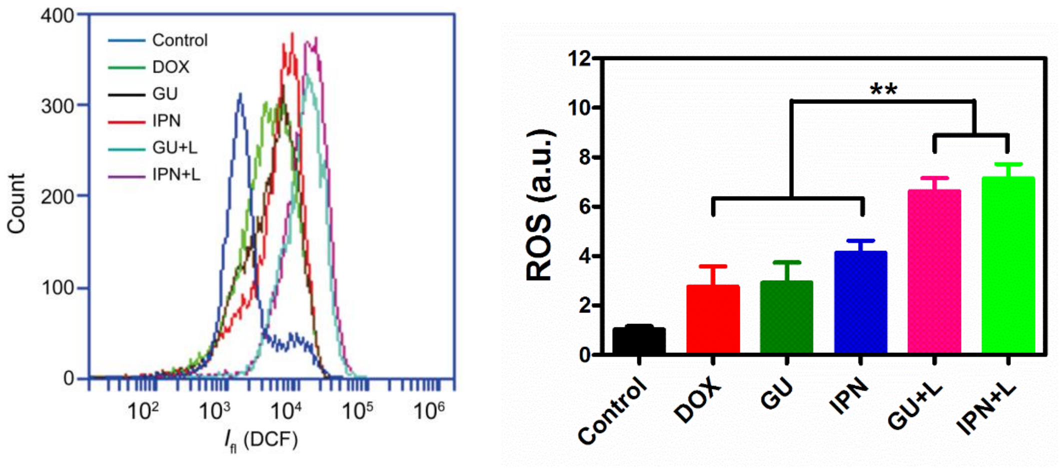

Figure S25. Intracellular ROS levels after treatment of 0.1% DMSO, DOX, GU, GU+L, IPN, and IPN+L, respectively.

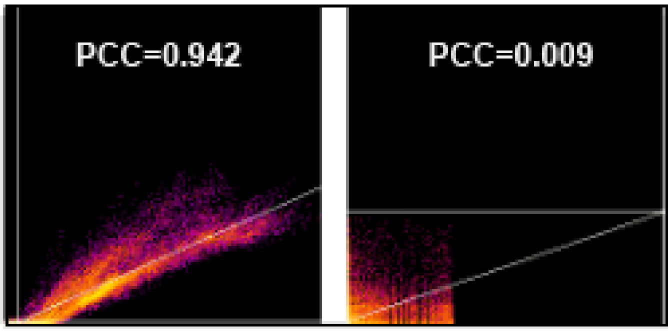

Figure S26. The quantitative analysis of mitochondria and Cyt C co-localization by Pearson's correlation coefficient (PCC) method.

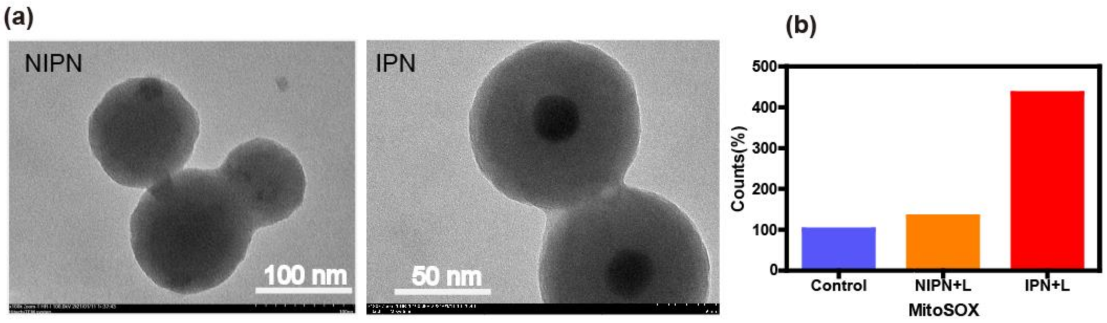

Figure S27. (a) TEM images of NIPNs and IPNs. (b) Mitochondrial superoxide level in control, NIPN+L, and IPN+L groups.

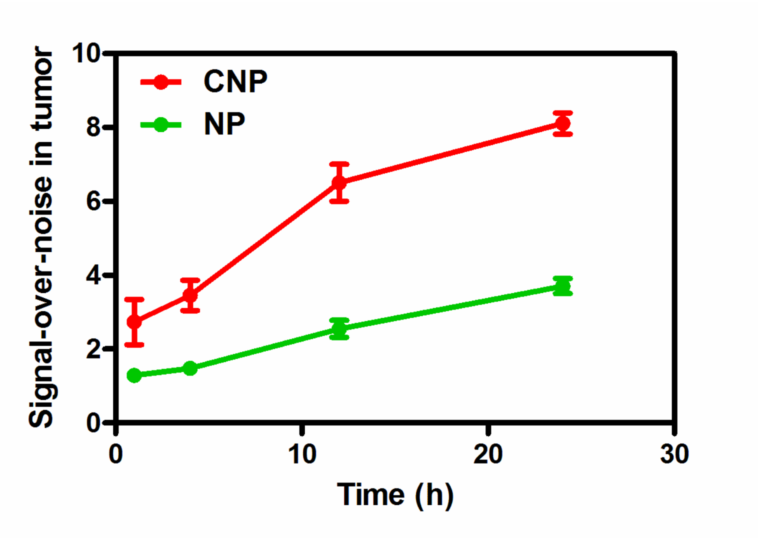

Figure S28. Signal-to-noise ratio in the tumors of CNP and NP groups at 1, 4, 12, and 24 h post-injection. Error bars indicate s.d. (n = 3).

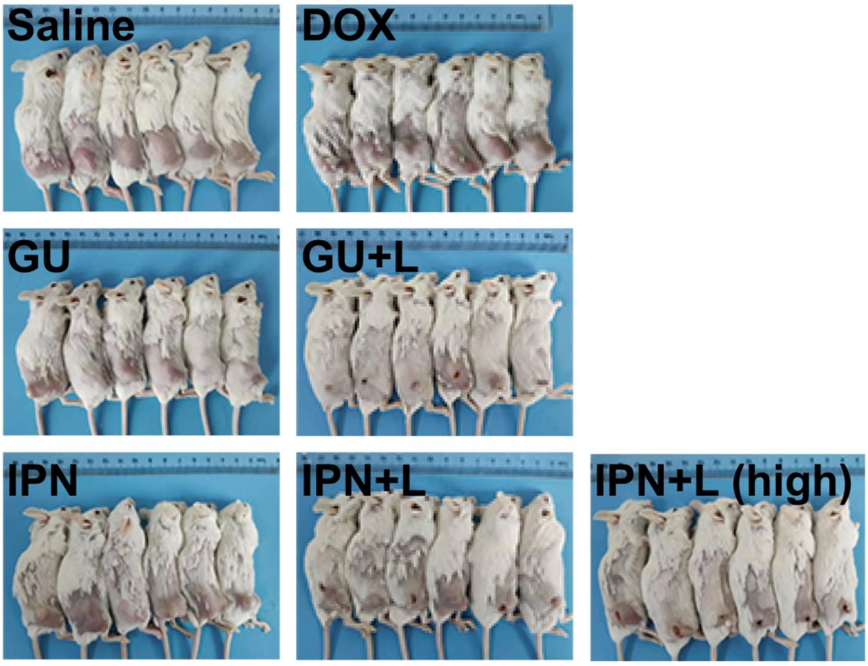

Figure S29. Photograph of 4T1 tumor-bearing mice after different treatments.

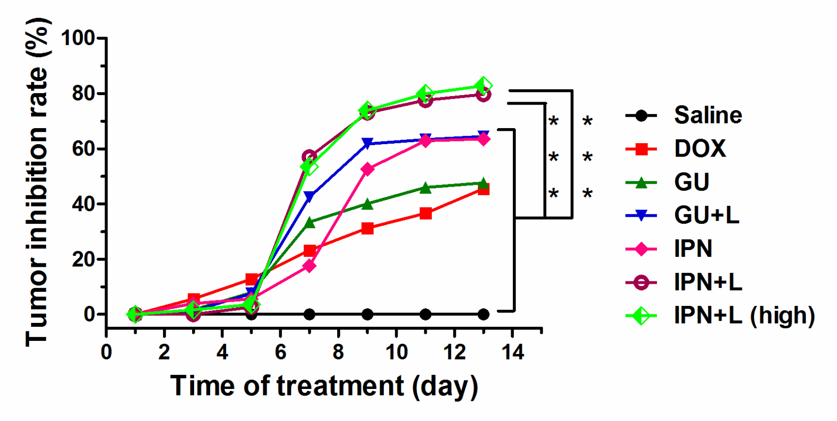

Figure S30. Tumor inhibition rate changes of 4T1 tumor-bearing mice during different treatments. Error bars indicate s.d. (n = 6). ****P* < 0.001.

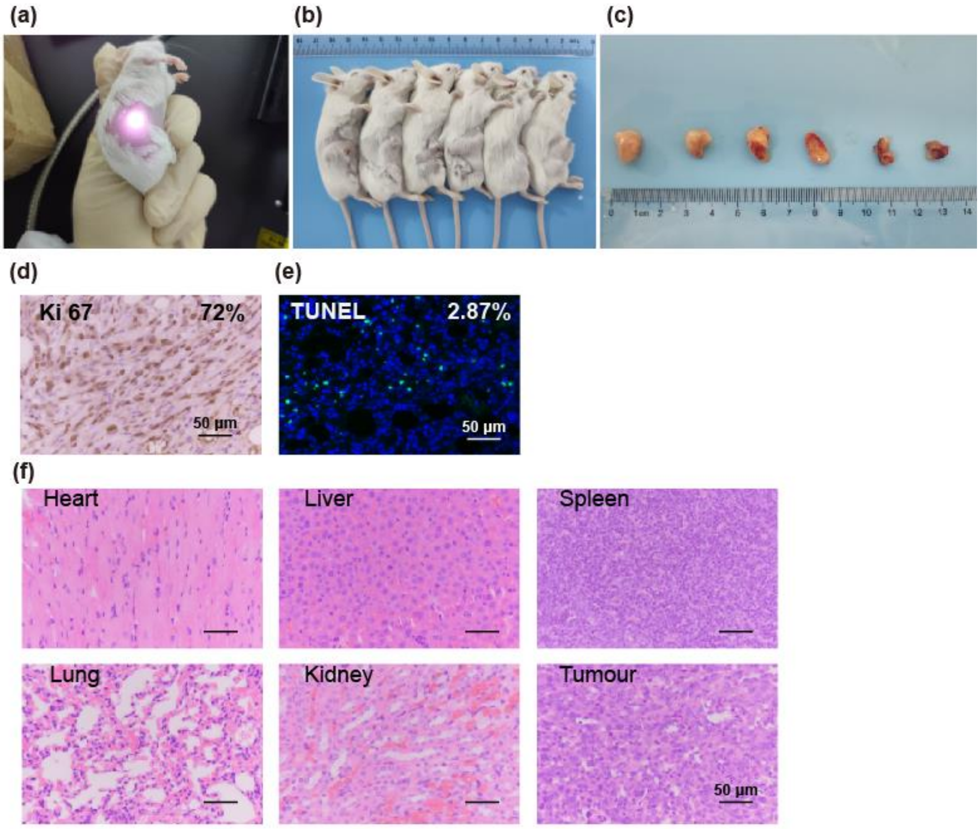

Figure S31. 4T1 tumor-bearing mice treated with 980 nm laser irradiation. (a) Photograph of 4T1 tumor-bearing mice under NIR irradiation. (b) Photograph of 4T1 tumor-bearing mice after 14-day treatment. (c) Images of the excised tumor tissues from tumor-bearing mice after treatment. (d) Images of the tumor tissues by Ki67 immunohistochemistry. (e) Detection of apoptosis in the tumor tissues by TUNEL assay. (f) Histological observation of the tumor tissues and major organs after staining with hematoxylin and eosin.

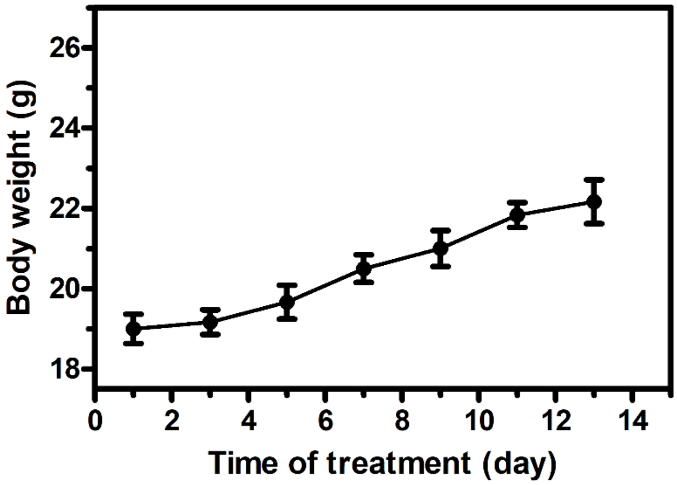

Figure S32. Body weight changes in tumor-bearing mice during 980 nm laser irradiation treatment. Error bars indicate the s.d. (n = 6).

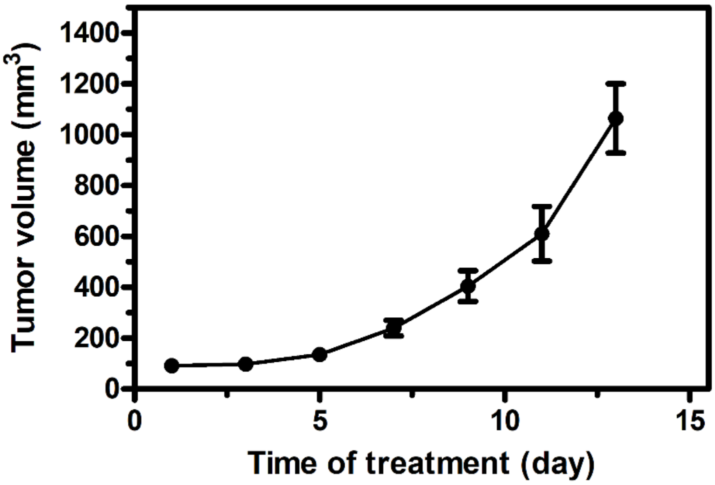

Figure S33. Tumor growth curves over 14 days after 980 nm laser irradiation treatment. Error bars indicate the s.d. (n = 6).

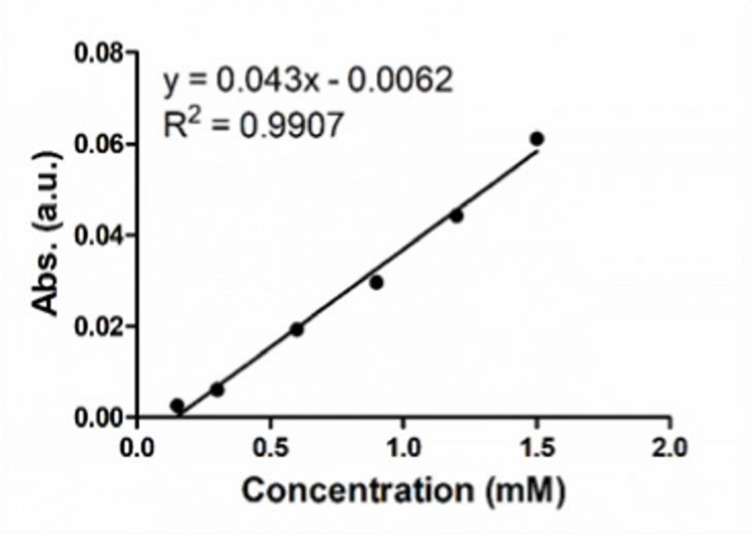

Figure S34. The standard curve of intratumoral total antioxygen capacity (T-AOC) assay.

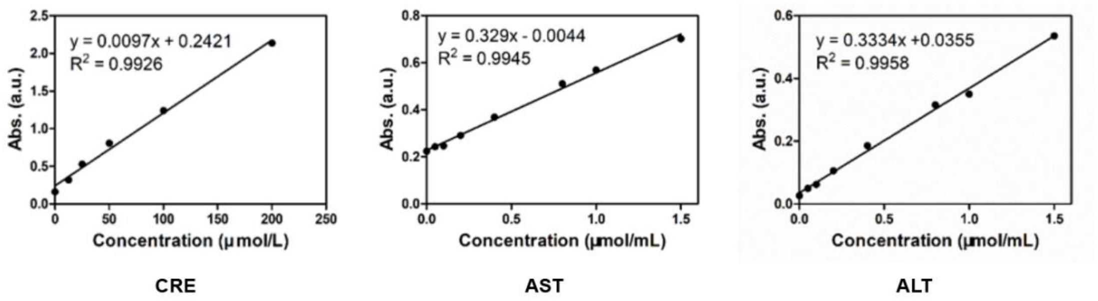

Figure S35. The standard curves of blood biochemistry analysis for CRE, AST, and ALT.
